# Infection of the maternal-fetal interface and vertical transmission following low-dose inoculation of pregnant rhesus macaques (*Macaca mulatta*) with an African-lineage Zika virus

**DOI:** 10.1371/journal.pone.0284964

**Published:** 2023-05-04

**Authors:** Michelle R. Koenig, Ann M. Mitzey, Terry K. Morgan, Xiankun Zeng, Heather A. Simmons, Andres Mejia, Fernanda Leyva Jaimes, Logan T. Keding, Chelsea M. Crooks, Andrea M. Weiler, Ellie K. Bohm, Matthew T. Aliota, Thomas C. Friedrich, Emma L. Mohr, Thaddeus G. Golos

**Affiliations:** 1 Department of Comparative Biosciences, University of Wisconsin-Madison, Madison, Wisconsin, United States of America; 2 Department of Pathology, Oregon Health and Science University, Portland, Oregon, United States of America; 3 Department of Obstetrics and Gynecology, Oregon Health and Science University, Portland, Oregon, United States of America; 4 Pathology Division, United States Army Medical Research Institute of Infectious Diseases, Frederick, MD, United States of America; 5 Wisconsin National Primate Research Center, University of Wisconsin-Madison, Madison, WI, United States of America; 6 Department of Obstetrics and Gynecology, University of Wisconsin-Madison, Madison, Wisconsin, United States of America; 7 Department of Pathobiological Sciences, University of Wisconsin-Madison, Madison, Wisconsin, United States of America; 8 Department of Veterinary and Biomedical Sciences, University of Minnesota, Twin Cities, St. Paul, Minnesota, United States of America; 9 Department of Pediatrics, University of Wisconsin-Madison, Madison, Wisconsin, United States of America; CEA, FRANCE

## Abstract

**Background:**

Congenital Zika virus (ZIKV) infection can result in birth defects, including malformations in the fetal brain and visual system. There are two distinct genetic lineages of ZIKV: African and Asian. Asian-lineage ZIKVs have been associated with adverse pregnancy outcomes in humans; however, recent evidence from experimental models suggests that African-lineage viruses can also be vertically transmitted and cause fetal harm.

**Methodology/Principal findings:**

To evaluate the pathway of vertical transmission of African-lineage ZIKV, we inoculated nine pregnant rhesus macaques (*Macaca mulatta*) subcutaneously with 44 plaque-forming units of a ZIKV strain from Senegal, (ZIKV-DAK). Dams were inoculated either at gestational day 30 or 45. Following maternal inoculation, pregnancies were surgically terminated seven or 14 days later and fetal and maternal-fetal interface tissues were collected and evaluated. Infection in the dams was evaluated via plasma viremia and neutralizing antibody titers pre- and post- ZIKV inoculation. All dams became productively infected and developed strong neutralizing antibody responses. ZIKV RNA was detected in maternal-fetal interface tissues (placenta, decidua, and fetal membranes) by RT-qPCR and *in situ* hybridization. *In situ* hybridization detected ZIKV predominantly in the decidua and revealed that the fetal membranes may play a role in ZIKV vertical transmission. Infectious ZIKV was detected in the amniotic fluid of three pregnancies and one fetus had ZIKV RNA detected in multiple tissues. No significant pathology was observed in any fetus; and ZIKV did not have a substantial effect on the placenta.

**Conclusions/Significance:**

This study demonstrates that a very low dose of African-lineage ZIKV can be vertically transmitted to the macaque fetus during pregnancy. The low inoculating dose used in this study suggests a low minimal infectious dose for rhesus macaques. Vertical transmission with a low dose in macaques further supports the high epidemic potential of African ZIKV strains.

## Introduction

Zika virus (ZIKV) is a flavivirus that is primarily transmitted by *Aedes spp*. mosquitoes [1]. ZIKV was initially discovered in Uganda in 1947 in a sentinel rhesus macaque (*Macaca mulatta*) [2]. ZIKV has since spread from the African continent to Asia, the Pacific, and the Americas [3]. Two distinct genetic lineages of ZIKV have been identified, African-lineage and Asian-lineage. Asian-lineage virus spread from Asia to the Pacific where it was detected in 2007 [3] and was introduced to Brazil between 2013 and 2014 [4]. The introduction of ZIKV in Brazil led to an outbreak in 2015 [4]. During this outbreak, ZIKV was first causally connected to birth defects and other adverse pregnancy outcomes [5].

*In utero* ZIKV exposure is associated with malformations in the fetal brain and visual system, along with fetal demise and pregnancy loss [6–12]. Of the two distinct genetic lineages of ZIKV, only Asian-lineage ZIKV is currently associated with congenital infection in humans [13]. Recently, African-lineage ZIKV has been reportedly detected in Brazil [Preprints 14, 15], however these data have yet to be published. The potential novel detection of African-lineage ZIKV outside the African continent demonstrates the possibility of a new outbreak and underscores the need to further evaluate the threat that African-lineage ZIKV poses to pregnant people.

Congenital birth defects or adverse pregnancy outcomes have not been formally associated with African-lineage ZIKV infection during pregnancy in people. However, *in vivo* studies done in mice and macaques suggest that African-lineage ZIKV could pose a substantial risk when infection occurs during pregnancy [16–19]. African-lineage infection in pregnant immune-deficient mice revealed the potential for vertical transmission during pregnancy [16, 17]. In contrast to Asian-lineage ZIKV, African-lineage ZIKV infection in pregnant mice is more likely to cause fetal demise than birth defects [16, 17]. Aubry et al. reported higher rates of fetal reabsorption in mouse pregnancies infected with African-lineage ZIKV, whereas infection with Asian-lineage resulted in varying capacities to cause fetal harm including fetal brain abnormalities as well as fetal death/resorption [16, 20].

The rhesus macaque model accurately reflects many key aspects of ZIKV infection during human pregnancy, thus making it a highly translatable model. Previous macaque studies have offered unique insights into the potential of African ZIKV infection to cause fetal harm during pregnancy [18, 19, 21]. These studies have all used the same low-passage African-lineage ZIKV strain from Senegal (ZIKV-DAK) yet have used a range of inoculation doses and routes, and gestational timing of infection. Crooks et al. found that a subcutaneous maternal inoculation of 1 x 10^4^ plaque forming units (PFU) at gestation day (gd) 45 resulted in a higher viral burden in the placenta than the same dose of an Asian-lineage virus [18], but did not find any evidence of vertical transmission at term. Newman et al. showed that maternal vaginal inoculation with 3 x 10^8^ PFU at gd 30 resulted in vertical transmission and fetal loss in two of three pregnancies [19]. Raasch et al. demonstrated that a maternal subcutaneous dose of 1 x 10^8^ PFU at gd 45 resulted in vertical transmission and fetal demise in three out of eight pregnancies [21]. The Crooks et al. and Raasch et al. studies suggest that vertical transmission may be dose-dependent. However, these studies evaluated the fetuses/neonates and maternal-fetal interface (MFI) tissues either when fetal death was detected or at gestational term, more than 15 weeks after maternal ZIKV inoculation.

Collectively, these data underscore the potential of African-lineage ZIKV to adversely affect the fetus *in utero*. To further evaluate the pathway of vertical transmission of African-lineage ZIKV from the mother to the fetus, we inoculated nine pregnant rhesus macaques with a relatively low dose of 44 PFU of ZIKV-DAK and terminated the pregnancies within two weeks post-inoculation. This design allows us to capture the early stages of infection in the MFI and vertical transmission and evaluate their vulnerability to African-lineage ZIKV. Furthermore, assessing the fetuses within two weeks of maternal ZIKV inoculation allowed us to investigate viral burden and pathology in the fetuses before fetal loss is likely to occur. This unconventional inoculation dose was the result of a dilution error, and since all dams became infected and developed viremia, this error was not detected before a total of nine animals were infected. Although our experimental design was not fully intentional, we believe these results are important and illuminating. We found that this low dose of African-lineage ZIKV is capable of infecting macaques and causing vertical transmission. This study indicates a low minimal infectious dose for this strain of African-lineage ZIKV in rhesus macaques and may suggest a high epidemic potential of African ZIKV strains.

## Materials and methods

### Experimental design

A total of nine pregnant female rhesus macaques (*Macaca mulatta*) were subcutaneously inoculated with 44 PFU of a Senegal isolate of African-lineage Zika virus ZIKV/*Aedes africanus*/SEN/DAK-AR-41524/1984 (ZIKV-DAK), Genbank accession number KY348860, during early pregnancy. This inoculation dose occurred due to an error in calculating the dilution factor for the viral inoculum due to an incorrect assumption that the stock of virus being used had a much higher titer. This dilution error occurred at the beginning of the study and was propagated over the set of animals reported here, thus this series of animals received a lower viral dose than was originally intended.

Five dams were infected at approximately gd 30, two of these pregnancies were terminated seven days post-infection (dpi), and three were terminated 14 dpi. Four dams were infected 15 days later in pregnancy, at approximately gd 45 followed by pregnancy termination at 14 dpi. When the dilution error was discovered, we did not continue to inoculate dams with 44 PFU/mL, thus the resulting study has uneven numbers in each group. A total of seven pregnancies terminated between gd 37 and gd 61 were used for controls for histological evaluation. Dams with control pregnancies were inoculated with saline and subjected to the same experimental regimen as the ZIKV infected pregnancies. Details on the timing of ZIKV/saline inoculation and pregnancy termination are provided in S1 Table.

### Ethics

The rhesus macaques used in this study were cared for by the staff at the Wisconsin National Primate Research Center (WNPRC) according to regulations and guidelines of the University of Wisconsin Institutional Animal Care and Use Committee, which approved this study protocol (G005691) in accordance with recommendations of the Weatherall report and according to the principles described in the National Research Council’s Guide for the Care and Use of Laboratory Animals. All animals were housed in enclosures with at least 4.3, 6.0, or 8.0 sq. ft. of floor space, measuring 30, 32, or 36 inches high, and containing a tubular PVC or stainless steel perch. Each individual enclosure was equipped with a horizontal or vertical sliding door, an automatic water lixit, and a stainless steel feed hopper. All animals were fed using a nutritional plan based on recommendations published by the National Research Council. Twice daily, macaques were fed a fixed formula, extruded dry diet (2050 Teklad Global 20% Protein Primate Diet) with adequate carbohydrate, energy, fat, fiber (10%), mineral, protein, and vitamin content. Dry diets were supplemented with fruits, vegetables, and other edible foods (e.g., nuts, cereals, seed mixtures, yogurt, peanut butter, popcorn, marshmallows, etc.) to provide variety to the diet and to inspire species-specific behaviors such as foraging. To further promote psychological well-being, animals were provided with food enrichment, human-to-monkey interaction, structural enrichment, and manipulanda. Environmental enrichment objects were selected to minimize chances of pathogen transmission from one animal to another and from animals to care staff. While on study, all animals were evaluated by trained animal care staff at least twice daily for signs of pain, distress, and illness by observing appetite, stool quality, activity level, and physical condition. Animals exhibiting abnormal presentation for any of these clinical parameters were provided appropriate care by attending veterinarians.

### Care & use of macaques

The female macaques described in this report were co-housed with a compatible male and observed daily for menses and breeding. Pregnancy was detected by abdominal ultrasound, and gestational age was estimated as previously described [22].

For physical examinations, virus inoculations, and blood collections, dams were anesthetized with an intramuscular dose of ketamine (10 mg/kg). Dams that were euthanized were first anesthetized with an intramuscular dose of ketamine (at least 15 mg/kg) followed by an intravenous overdose (greater than or equal to 50 mg/kg or to effect) of sodium pentobarbital. Blood samples from the femoral or saphenous vein were obtained using a vacutainer system or needle and syringe. Pregnant macaques were monitored daily prior to and after inoculation for any clinical signs of infection (e.g., diarrhea, inappetence, inactivity, and atypical behaviors) and general well-being.

### Viral inoculate preparation and PFU calculation

Nine pregnant dams were inoculated subcutaneously with 44 PFU of ZIKV/*Aedes africanus*/SEN/DAK-AR-41524/1984 (ZIKV-DAK). This virus was originally isolated from *Aedes africanus* mosquitoes with a round of amplification on *Aedes pseudocutellaris* cells, followed by amplification on C6/36 cells and two rounds of amplification on Vero cells. ZIKV-DAK was obtained from BEI Resources (Manassas, VA). The stock of ZIKV-DAK was evaluated using plaque assay on Vero cells as previously described [18]. The original titer of the viral stock was determined to be 3.2 x 10^6^ PFU/mL.

All dams were inoculated with a 1:7.3 x 10^4^ dilution of the viral stock, prepared in an identical fashion. Based on this dilution factor, we calculate that the animals received a dose of 44 PFU. After the dilution error was discovered, we thawed another vial of the virus stock and repeated plaque assays using the same method as described above, and determined the titer to be 3.4 x 10^6^ PFU/mL, a value highly similar to the original titer. This finding indicates that viral infectivity was stable over time in cryostorage and supports the conclusion that the animals studied here received a calculated dose of ranging from 43.84–46.58 PFU.

The dilution of the viral stock in sterile PBS was done in two steps. First, a vial of the stock frozen at 3.2 x 10^6^ PFU/mL was thawed. After thawing, 1.36 x 10^−2^ mL of the virus stock (4.352 x 10^4^ PFU in 13.6 μL) was added to 9.986 mL of 1X PBS creating a 10 mL solution of 4.352 x 10^3^ PFU/ 1mL. Next, 1.0 x 10^−1^ mL of the diluted stock created in step one was added to 9.9 mL of PBS resulting in a 10mL solution with a titer 4.354 x 10^1^ PFU/mL. 1mL of that the final diluted stock (43.54 PFU/1mL) was loaded into a syringe and kept on ice until inoculation. The stock was diluted in this manner because it was incorrectly believed by those preparing the stock that the titer of the frozen viral stock was 7.3 x 10^8^ PFU/mL rather than 3.2 x 10^6^.

### Inoculation and monitoring

Animals were anesthetized as described above, and 1 mL of the inoculum (43.54 PFU/1mL) described in the previous subsection was delivered subcutaneously over the cranial dorsum. Animals were monitored closely following inoculation for any signs of an adverse reaction. Seven pregnant dams served as controls and were inoculated subcutaneously with 1mL of sterile PBS, using the same procedure as described above. All control animals were subjected to the same sedation and blood collection schedule as the ZIKV inoculated animals.

### Pregnancy termination and tissue collection

A total of 16 dams had their pregnancies surgically terminated and products of conception collected at gd 36–63 via uterotomy. This procedure was terminal for three dams, 45/14-4, 30/14-1 and 30/14/-C1, per IACUC protocol specifications unrelated to viral infection and thus plasma viremia and PRNT data points are not provided for 45/14-4 and 30/14-1 beyond 14 dpi. During the uterotomy procedure, the entire conceptus (fetus, placenta, fetal membranes, umbilical cord, and amniotic fluid) was removed. The tissues for all animals were dissected using sterile instruments which were changed between each organ/tissue to minimize possible cross-contamination. Each organ/tissue was evaluated grossly *in situ*, removed with sterile instruments, placed in a sterile culture dish, and dissected for histology, viral burden assay, or banked for future assays. Samples of the MFI included two full-thickness center-cut sections of the primary and secondary placental discs containing decidua basalis and chorionic plate, these center cuts were approximately 5–10 mm wide depending on the size of the placenta. Biopsy samples were collected for RT-qPCR evaluation including: decidua basalis dissected from the maternal surface of the placenta, chorionic plate dissected away from the placental disc, amniotic membrane, chorionic membrane, placental bed (the uterine placental attachment site containing deep decidua basalis and uterine myometrium), and biopsies from the body of the placenta. To assess viral burden via RT-qPCR three placental biopsies were taken of approximately 3–5 mm in width were taken from each placental disc: one near the placental margin, one midway between the margin and the center, and one near the center of the placenta. Placental biopsies were taken after the decidual basalis was removed; however histological evaluation of the samples taken in the same manner in subsequent studies reveal that there are often portions of the trophoblastic shell on these placental samples. Additional samples from the placenta, decidua, chorionic plate, and fetal membranes (amnion and chorion) were also collected for future assays. Fetal tissues and fluids collected for viral burden assay, or banked for future assays included; cerebral spinal fluid (CSF), fetal brain with or without skull, eye, heart or fetal chest, fetal limb containing muscle and skin, liver, kidney, and spleen. The remaining fetal tissues were preserved for histological evaluation. CSF was collected from the cisterna magna at the atlanto-occiptal joint with a 27 gauge needle and syringe, volumes ranged from 0.2 to 1.5 mL. To collect umbilical cord blood the cord was held with forceps, transected, and the cut end of the cord was placed in a blood collection tube, collecting 20–100 μL of blood. The extraembryonic celoemic fluid was not recovered during the collection of amniotic fluid (typically between 1–6 mL).

### Viral RNA isolation from blood, tissues, and other fluids

RNA was isolated from maternal and fetal plasma, CSF, and amniotic fluid using the Viral Total Nucleic Acid Purification Kit (Promega, Madison, WI) on a Maxwell 48 RSC instrument (Promega, Madison, WI) as previously reported [23]. Fetal and maternal tissues were processed with RNAlater (Invitrogen, Carlsbad, CA) according to the manufacturer’s protocols. RNA was recovered from tissue samples using a modification of a previously described method [24]. Briefly, up to 200 mg of tissue was disrupted in TRIzol (Life Technologies, Carlsbad, CA) with 2 x 5 mm stainless steel beads using a TissueLyser (Qiagen, Germantown, MD) for 3 minutes at 25 r/s for 2 cycles. Following homogenization, samples in TRIzol were separated using Bromo-chloro-propanol (Sigma-Aldrich, St. Louis, MO). The aqueous phase was collected, and glycogen was added as a carrier. The samples were washed in isopropanol and ethanol precipitated. RNA was re-suspended in 5 mM Tris pH 8.0 and stored at -80°C.

### Quantitative reverse transcriptase (RT-qPCR)

ZIKV RNA was isolated from both fluid and tissue samples as previously described [17]. Viral RNA was then quantified using a highly sensitive RT-qPCR assay based on the one developed by Lanciotti et al. [25], though the primers were modified with degenerate bases at select sites to accommodate African-lineage Zika viruses. RNA was reverse-transcribed and amplified using the TaqMan Fast Virus 1-Step Master Mix RT-qPCR kit (Life Technologies, Carlsbad, CA) on the LightCycler 480 or LC96 instrument (Roche, Indianapolis, IN), and quantified by interpolation onto a standard curve made up of serial tenfold dilutions of *in vitro* transcribed RNA. RNA for this standard curve was transcribed from a plasmid containing an 800 bp region of the Zika virus genome that is targeted by the RT-qPCR assay. The final reaction mixtures contained 150 ng random primers (Promega, Madison, WI), 600 nM each primer and 100 nM probe. Primer and probe sequences are as follows: forward primer: 5’- CGYTGCCCAACACAAGG-3’, reverse primer: 5′-CCACYAAYGTTCTTTTGCABACAT-3′ and probe: 5′-6-carboxyfluorescein-AGCCTACCTTGAYAAGCARTCAGACACYCAA. The limit of detection for fluids (plasma, amniotic fluid, CSF) with this assay is 150 copies/ml. The theoretical limit of detection for the tissues is 3 copies/mg [18].

### Plaque reduction neutralization test (PRNT)

Macaque serum samples were assessed for ZIKV neutralizing antibodies utilizing a plaque reduction neutralization test (PRNT). Endpoint titrations of reactive sera, utilizing a 90% cutoff (PRNT_90_), were performed as previously described [26] against ZIKV/Aedes africanus/SEN/DAK-AR-41524/1984 (ZIKV-DAK). Briefly, ZIKV was mixed with serial 2-fold dilutions of serum for 1 hour at 37°C before being added to Vero cells, and neutralization curves were generated using GraphPad Prism software (La Jolla, CA). The resulting data were analyzed by nonlinear regression to estimate the dilution of serum required to inhibit both 90% and 50% of infection.

### Viral quantification by plaque assay

Amniotic fluid samples from 30/14-1, 30/7-2, 45/14-4, and 30/14-3 were evaluated for infectious ZIKV by plaque assay. These samples were selected based on RT-qPCR results showing a high level of ZIKV RNA. Titrations for replication competent virus quantification of amniotic fluid was completed by plaque assay on Vero cell cultures as described previously [27]. Vero cells were obtained from the American Type Culture Collection (CCL-81). Duplicate wells were infected with 0.1 mL of aliquots from serial 10-fold dilutions in growth media and virus was adsorbed for 1h. Following incubation, the inoculum was removed, and cell monolayers were overlaid with 3 mL containing a 1:1 mixture of 1.2% oxoid agar and DMEM (Gibco, Carlsbad, CA, USA) with 10% (vol/vol) FBS and 2% (vol/vol) penicillin/streptomycin. Cells were incubated at 37˚C in 5% CO_2_ for 2 days for plaque development. Cell monolayers were then stained with 3 mL of overlay containing a 1:1 mixture of 1.2% oxoid agar and DMEM with 2% (vol/vol) FBS, 2% (vol/vol) penicillin/streptomycin and 0.33% neutral red (Gibco). Cells were incubated overnight at 37˚C and plaques were counted. Limit of detection for the plaque assay is 0.7 log10 PFU/mL.

### Histology

To assess general pathology, tissues were fixed in 4% PFA, routinely processed, and embedded in paraffin. Paraffin sections (5 μm) were stained with hematoxylin and eosin (H&E) or used for *in situ* hybridization (ISH). Histological evaluation of fetal tissue was done by a board-certified veterinary pathologist (HAS, AM) blinded to the ZIKV RNA results. Histologic examination of placental sections was performed by an experienced placental pathologist (TKM) who was blinded to ZIKV RNA results and pregnancy treatment, but was informed of the gestational age at the time of collection to properly date villous maturation. Uteroplacental histologic sections were scored for the presence or absence of leukocytoclastic decidual spiral artery vasculitis, patchy decidual necrosis (excluding fibrinoid layer between anchoring villi and maternal decidua), chorionic villous infarctions (acute or chronic), and villous stromal calcifications. Photomicrographs were taken using brightfield on the Nikon eclipse Ti2 (Nikon Instruments Inc., Melville, NY, USA) using NIS-Elements AR software version 5.02.006 (Nikon Instruments Inc., Melville, NY, USA). Scale bars were added using NIS-Elements AR and photomicrographs were white balanced using Adobe Photoshop 2020 version 21.10 (Adobe Inc, San Jose, CA, USA).

### Detection of ZIKV RNA using *in situ* hybridization (ISH)

ISH was conducted as previously described [22]. The ISH probes against the Zika virus genome were purchased commercially (Advanced Cell Diagnostics, Cat No. 468361, Newark, California, USA). Tissues/structures were determined to be negative when ZIKV RNA was undetectable by RNAscope ISH, including instances when only a single focus of staining was observed. Tissues/structures were determined to be positive for ZIKV RNA as detectable by RNAscope ISH when there was clear staining across a tissue/structure; any positive signal that was limited to a single cell-sized focus of indeterminate histological appearance (granular “specks” of positive signal) was interpreted as non-specific staining. Uninfected tissue sections served as negative controls. An example of the negative control and as well as examples of non-specific staining can be seen in S1 Fig.

### Detection of ZIKV replication using multiplex fluorescence *in situ* hybridization (mFISH)

Multiplex fluorescence *in situ* hybridization (mFISH) was performed using the RNAscope® Fluorescent Multiplex Kit (Advanced Cell Diagnostics, Newark, CA) according to the manufacturer’s instructions with modifications. Twenty ZZ probe pairs with C1 channel (green, Cat# 463781) targeting ZIKV positive sense RNA and forty ZZ probe pairs with C3 channel (red, Cat# 467911) targeting ZIKV negative sense RNA are synthesized by Advanced Cell Diagnostics. Formalin-fixed paraffin-embedded (FFPE) tissue sections were deparaffinized using xylene and hydrated through a series of ethanol washes. These tissue sections were further heated in the antigen retrieval buffer and digested by proteinase. Sections were exposed to ISH target probes and incubated at 40°C in a hybridization oven for two hours. After rinsing, ISH signal was amplified using company-provided Pre-amplifier and Amplifier conjugated to fluorescent dye. Sections were counterstained with 4’, 6-diamidino-2-phenylindole (DAPI, Thermo Fisher Scientific, Waltham, MA, USA), mounted, and stored at 4°C until image analysis. FISH images were captured on an LSM 880 Confocal Microscope with Airyscan (Zeiss, Oberkochen, Germany) and processed using open-source ImageJ software (National Institutes of Health, Bethesda, MD, USA). Uninfected tissue sections served as negative controls.

### Immunohistochemistry

CD56, CD68, and CD31 stained tissues sections were immunostained using the Benchmark XT autostainer (Ventana Medical Systems, Tucson, AZ, USA) using provided pre-diluted antibodies CD56, CD68, and CD31 (Ventana Medical Systems). For cytokeratin and CD163 staining immunostaining, sections were deparaffinized, incubated in 3% hydrogen peroxide for 15 min, and subjected to a heat induced antigen retrieval protocol (10 mM citric acid at 110°C for 15 min). Sections were immunohistochemically stained using a proprietary polymer-based peroxidase staining method (Biocare Medical, Concord, CA): sections were blocked 20 min in Background Punisher (Biocare Medical) and incubated in 1:100 mouse anti-cytokeratin CAM5.2 monoclonal antibody (item 452M-94, Sigma-Aldrich, St. Louis, MO) or 1:1000 anti-CD163 monoclonal antibody (Novus Biologicals, Centennial, CO. Ref NB110-40686) overnight at 4 degrees. Slides were processed for bound antibody detection by incubation in full-strength MACH2 Polymer-horseradish peroxidase conjugate (Biocare Medical) for 20 min and developed with Betazoid DAB chromogen (Biocare Medical) for 1.5 min. All washes were in 0.1 M Tris pH 7.5 with Tween-20 at room temperature.

### Statistical analysis

All statistical analysis was conducted using Graphpad Prism 9 for macOS version 9.3.1 software. Unpaired Student’s t-test was used to determine significant differences in the timing of peak viremia, and viral burden at peak viremia. Fisher’s exact test was used to compare the presence or absence of decidual vasculitis in mock vs ZIKV-DAK placentas. Fisher’s exact test was also used to evaluate the presence or absence of decidual necrosis in mock vs ZIKV-DAK placentas.

## Results

### Low-dose ZIKV-DAK inoculation resulted in productive infection and neutralizing antibody responses in all dams

**Fig 1:**
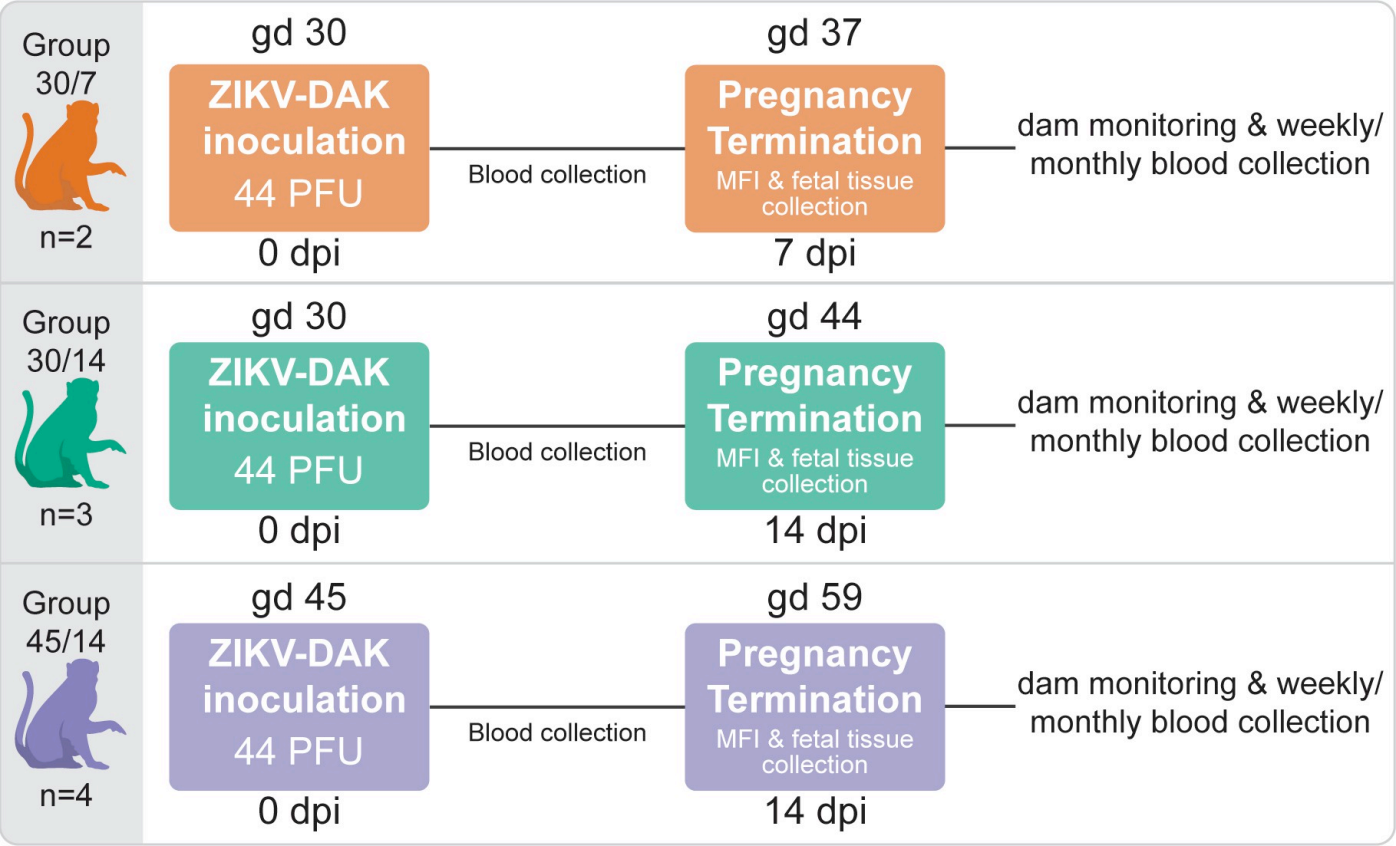
The effect of ZIKV-DAK on pregnancy was evaluated in three groups of pregnant rhesus macaques. Three groups of pregnant macaques were inoculated early in gestation with 44 PFU of ZIKV-DAK at either gd 30 (Group 30/7 and Group 30/14) or gd 45 (Group 45/14). Viremia in the dams was monitored via blood collection. Pregnancies were surgically terminated at either 7 dpi (Group 30/7) or 14 dpi (Group 30/14 and Group 45/14) and MFI and fetal tissues were collected for evaluation. After pregnancy termination, the dams were monitored and blood was collected and evaluated weekly until ZIKV was no longer detected in the blood. When ZIKV was no longer detected in the blood for two consecutive blood draws, the dam was switched to monthly blood collection through 87 dpi.

A total of nine pregnant rhesus macaques were subcutaneously inoculated with a Senegalese isolate of African-lineage ZIKV during early pregnancy (Fig 1), when the fetus is at greatest risk of congenital ZIKV infection and associated birth defects [7, 9, 11, 28, 29]. Five dams were infected at approximately gd 30 and four dams at approximately gd 45. Blood was collected from the dams prior to infection, during the first two weeks after inoculation, and intermittently until up to 87 dpi. All dams received a dose of 44 PFU; although this dose was not intentionally chosen, all dams became productively infected and developed neutralizing antibody responses. Physical examinations of infected dams found no ZIKV-associated symptoms reported in human infections such as rash or conjunctivitis following inoculation. This finding is consistent with previous reported studies of ZIKV-DAK infection in rhesus macaques [21].

**Fig 2:**
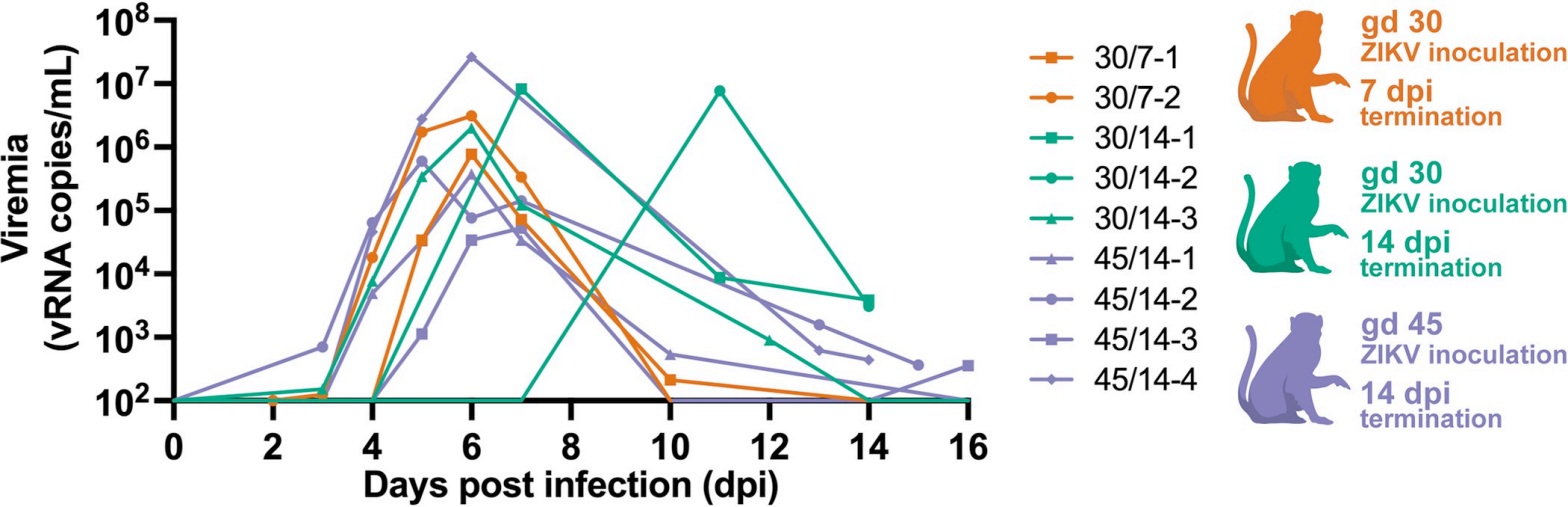
Plasma viremia following inoculation with 44 PFU of ZIKV-DAK. Plasma ZIKV loads were determined by RT-qPCR and are represented as vRNA copies/mL from 0 up to 16 dpi. Only values above the limit of detection of 150 copies/mL are shown. Experimental groups are represented by different colors, symbols represent individual animals within experimental groups.

All dams developed plasma viremia: seven animals resolved viremia between 10 and 16 dpi, and two dams had positive plasma viremia at 14 dpi when they were euthanized (Fig 2). Peak plasma viremia levels were between 5 x 10^4^ and 2.65 x 10^7^ ZIKV RNA copies/mL and peaked between 6 and 11 dpi. By comparing these results to a previous report that inoculated pregnant rhesus macaques with a larger dose (10^4^ PFU) of the same ZIKV isolate at gd 45 [18], we see that the animals given 44 PFU had a significant delay (p = 0.0150) in time to peak plasma viremia (S2A Fig), but reached a similar peak level of virus in the blood (S2B Fig).

**Fig 3:**
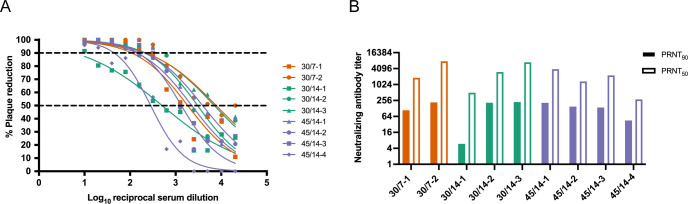
Neutralizing antibody titers following inoculation with 44 PFU of ZIKV-DAK. Plaque reduction neutralization tests (PRNT) were performed on serum samples collected at 14 dpi (45/14-4 and 30/14-1) or approximately 28 dpi (all other animals). Data are expressed relative to infectivity in the absence of serum. A) Neutralization curves. B) PRNT_90_ and PRNT_50_ values were estimated using nonlinear regression analysis and are indicated with dotted lines in Panel A.

To determine if infection with 44 PFU ZIKV was sufficient to induce neutralizing antibody (nAb) responses, we measured nAb titers in the serum prior to infection and at approximately 14 or 28 dpi. All animals had robust titers of nAb at approximately 14 or 28 dpi (Fig 3): the titers of nAb induced by inoculation with 44 PFU are not significantly different from those induced by challenge with 10^4^ PFU [18] (S3 Fig). One animal, 45/14-1, had low titers of nAb prior to infection (S4 Fig). 45/14-1 was not exposed to ZIKV or any other flavivirus prior to this study. It is not clear why she had antibodies that were neutralizing prior to ZIKV-DAK inoculation. However, we believe her data are still comparable to the other ZIKV naive dams in this study. Her PRNT_90_ titer at 0 dpi was only ~1:4, which is well below the threshold of what is considered in the field to be diagnostic of prior ZIKV infection [30]. Thus, we cannot robustly conclude that the neutralization response at 0 dpi is “ZIKV-specific” [31]. Furthermore, this animal developed viral load kinetics similar to other animals included in the study (Fig 2) and we observed a greater than four-fold rise in PRNT_90_ titers from 0 to 28 dpi.

### ZIKV-DAK was consistently detected in maternal-fetal interface tissues shortly after maternal inoculation

To assess the presence of ZIKV in the MFI we surgically terminated the pregnancies shortly after maternal inoculation. Two pregnancies were terminated at 7 dpi and seven pregnancies were terminated 14 dpi. Tissues and fluids from the MFI were evaluated for ZIKV RNA using RT-qPCR and ISH. Biopsies from the dam’s spleen, liver, and mesenteric lymph node were collected at the time of surgery. ZIKV RNA was found in at least one of the biopsied tissues for all but one dam (30/7-2) (S5A Fig).

**Fig 4:**
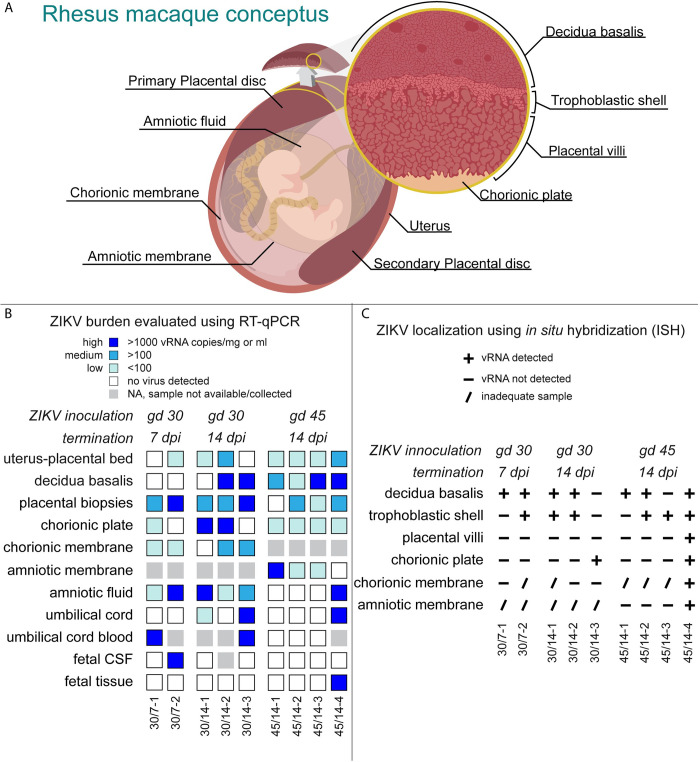
ZIKV-DAK detection in MFI and fetal tissues and fluids. A) Illustration of the rhesus macaque conceptus depicting evaluated tissues and fluids. The pregnant uterus is lined by the maternal endometrium of pregnancy called the decidua. The layers of the placenta are shown as the trophoblastic shell where the trophoblasts and the decidua meet, the placental villi, and the chorionic plate made up of the chorion and large fetal vessels. The chorionic membrane is the outer fetal membrane and the inner fetal membrane is the amniotic membrane. As the fetus grows the amniotic membrane is pushed up against the chorionic membrane and the membranes are fused. Fused fetal membranes were only observed in one case, 45/14-4. B) ZIKV RNA burden as detected by RT-qPCR on RNA isolated from indicated tissues and fluids. The level of ZIKV burden is summarized as high (>1000 ZIKV RNA copies/mg of tissue or mL of fluid), medium (>100 ZIKV RNA copies/mg or mL), low (< 100 ZIKV RNA copies/mg), or below the limit of detection. The limit of detection for all the fluids listed (CSF, amniotic fluid, umbilical cord blood) is 150 copies/mL. The theoretical limit of detection for all of the tissues is 3 copies/mg. Placental viral loads are presented as the mean of three tissue biopsies per disc. Placental biopsies represent a general survey of the placenta as these samples contain maternal blood, chorionic plate, and portions of the trophoblastic shell in addition to placental villi. C) ZIKV RNA presence as detected by ISH. ZIKV RNA was noted as present (+) or absent (-) in the indicated tissues. In some instances, a histological sample was not obtained, thus ZIKV RNA presence or absence could not be properly evaluated (/).

Collectively, ISH and RT-qPCR results show that the MFI was consistently infected with ZIKV, with virus present in the placenta, decidua basalis, amniotic membrane and chorionic membrane (Fig 4). Details on viral burden in MFI tissues are shown in S5B Fig. ZIKV burden in MFI tissues from all three experimental groups were similar (S5C Fig). ISH demonstrated that infection in the placenta was mainly confined to the trophoblastic shell and the chorionic plate. Scanned images of the positive ISH results can be found Dryad Digital Repository (https://doi.org/10.5061/dryad.931zcrjpg). ZIKV RNA was detected within placental villi in only one pregnancy (Fig 4C). Additionally, ISH evaluation showed that the decidua basalis and trophoblastic shell were the most frequently infected MFI tissue (Fig 4C). Random sampling bias may explain discrepancies between the RT-qPCR and ISH results in the decidua basalis, chorionic plate, and amniotic and chorionic membranes. Furthermore, ISH evaluation suggests that infection at this stage is often focal or in multiple small foci and not widespread throughout the tissues.

### Maternal inoculation with 44 PFU of ZIKV-DAK is sufficient to cause vertical transmission

**Fig 5:**
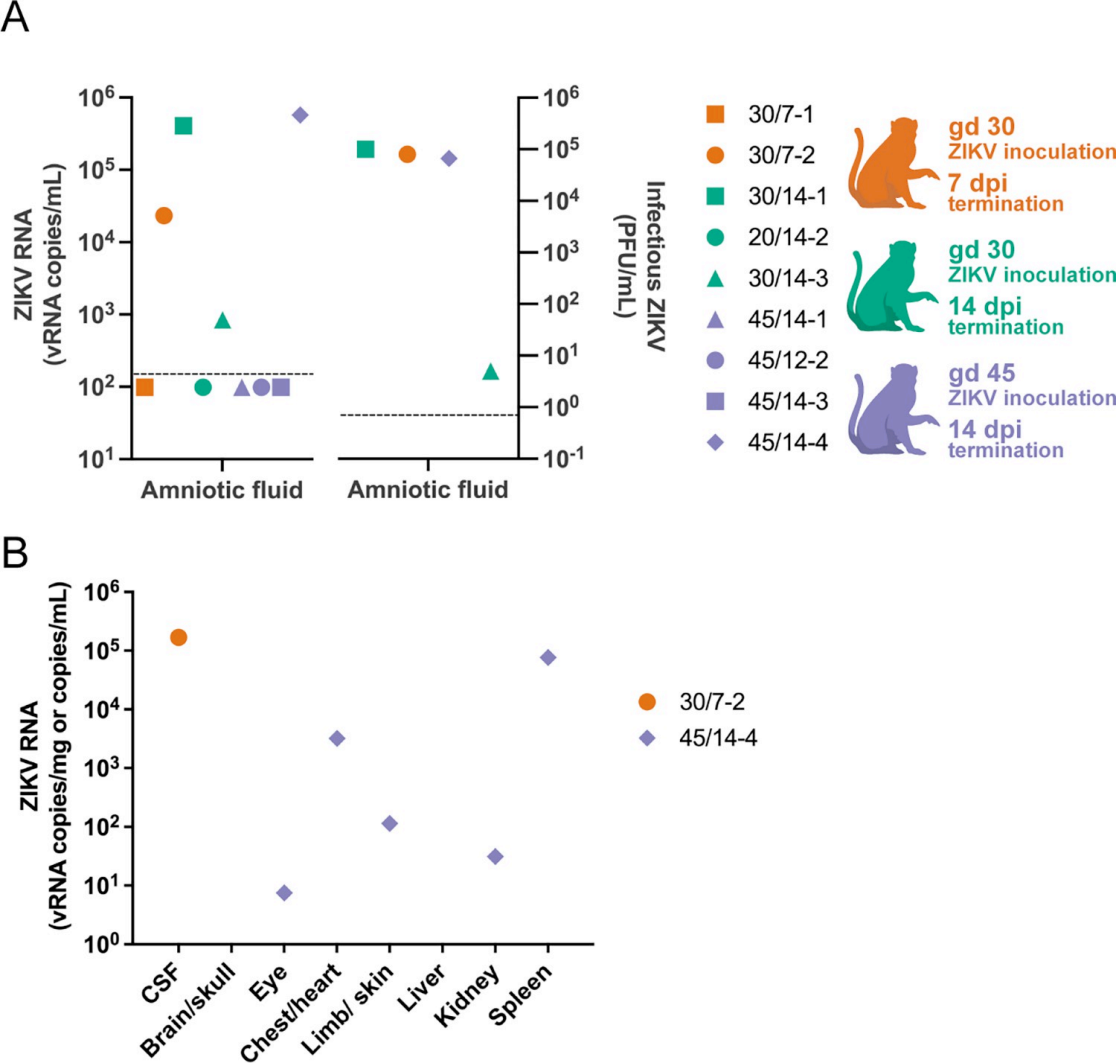
ZIKV burden in fetal tissues and fluids. A) Amniotic fluid ZIKV RNA levels determined by RT-qPCR (left panel) and infectious ZIKV determined by plaque assay (right panel). Limit of detection for the RT-qPCR fluid assay shown on the left is 150 copies/mL, this is represented by the dashed line. On the right, the dashed line represents the limit of detection of 0.7 PFU/mL for the plaque assay. Four amniotic fluid samples were analyzed by plaque assay based on positive detection of vRNA by RT-qPCR. B) ZIKV RNA isolated from fetal tissues and fluids; viral loads are measured in vRNA copies/mL of fluid (CSF) or vRNA copies/mg of tissue. Limit of detection for the RT-qPCR fluid assay (CSF) is 150 copies/mL and the theoretical limit of detection for the RT-qPCR assay for the tissues is 3 copies/mg. Only samples above the limit of detection are shown.

In this study, we defined vertical transmission as the presence of infectious virus in the amniotic fluid. To determine the capacity of a maternal inoculation of 44 PFU to achieve vertical transmission, we evaluated the amniotic fluid with RT-qPCR and then further assessed positive samples with a plaque assay. We found infectious ZIKV in three cases three (45/14-4, 30/14-1, 30/7-2) (Fig 5A), thus confirming vertical transmission in these cases. Additionally, ZIKV RNA was detected in the umbilical cord tissue in two cases and the umbilical cord blood in two cases (Fig 4B, S5D Fig), but we were unable to confirm that the ZIKV RNA found represented a replicating virus. In one case with vertical transmission, 45/14-4, ZIKV RNA was detected in multiple fetal tissues (Figs 4B and 5B). and in 30/7-2 ZIKV RNA was detected in the fetal CSF (Figs 4B and 5B). We were unable to confirm that RNA detected in the fetal tissues of 45/14-4 represented replicating virus, due to the absence of negative-sense ZIKV RNA as determined using mFISH. Although no replicating virus was detected in this fetus, the presence of infectious virus in the amniotic fluid confirms that ZIKV-DAK was vertically transmitted to the fetus. Maternal inoculation with 44 PFU at gd 30 versus gd 45 appeared to have no impact on the capacity for vertical transmission. Furthermore, pregnancies that were allowed to progress to 14 dpi compared to 7 dpi also did not demonstrate an increased likelihood of vertical transmission. Collectively, these data show that maternal inoculation of 44 PFU of ZIKV-DAK is sufficient to cause vertical transmission as early as 7 dpi.

### ZIKV-DAK may vertically transmit through the fetal membranes

To understand the pathway of vertical transmission, we used ISH to evaluate the cellular location of ZIKV RNA in histological specimens from cases that had infectious virus in the amniotic fluid (30/7-2, 30/14-1, and 45/14-4). For 30/7-2 and 30/14-1, ISH was performed on full-thickness placental center cuts taken from the primary and secondary placental discs which included decidua basalis, trophoblastic shell, placental villi, and chorionic plate with large fetal vessels. In both cases, ZIKV RNA was detected in the decidua basalis and trophoblast shell but not in the placental villi or chorionic plate (Fig 4C). Unfortunately, we were not able to obtain a high-quality histological specimen of either of the amniotic or chorionic membrane from these two cases to properly evaluate the presence of ZIKV RNA.

**Fig 6:**
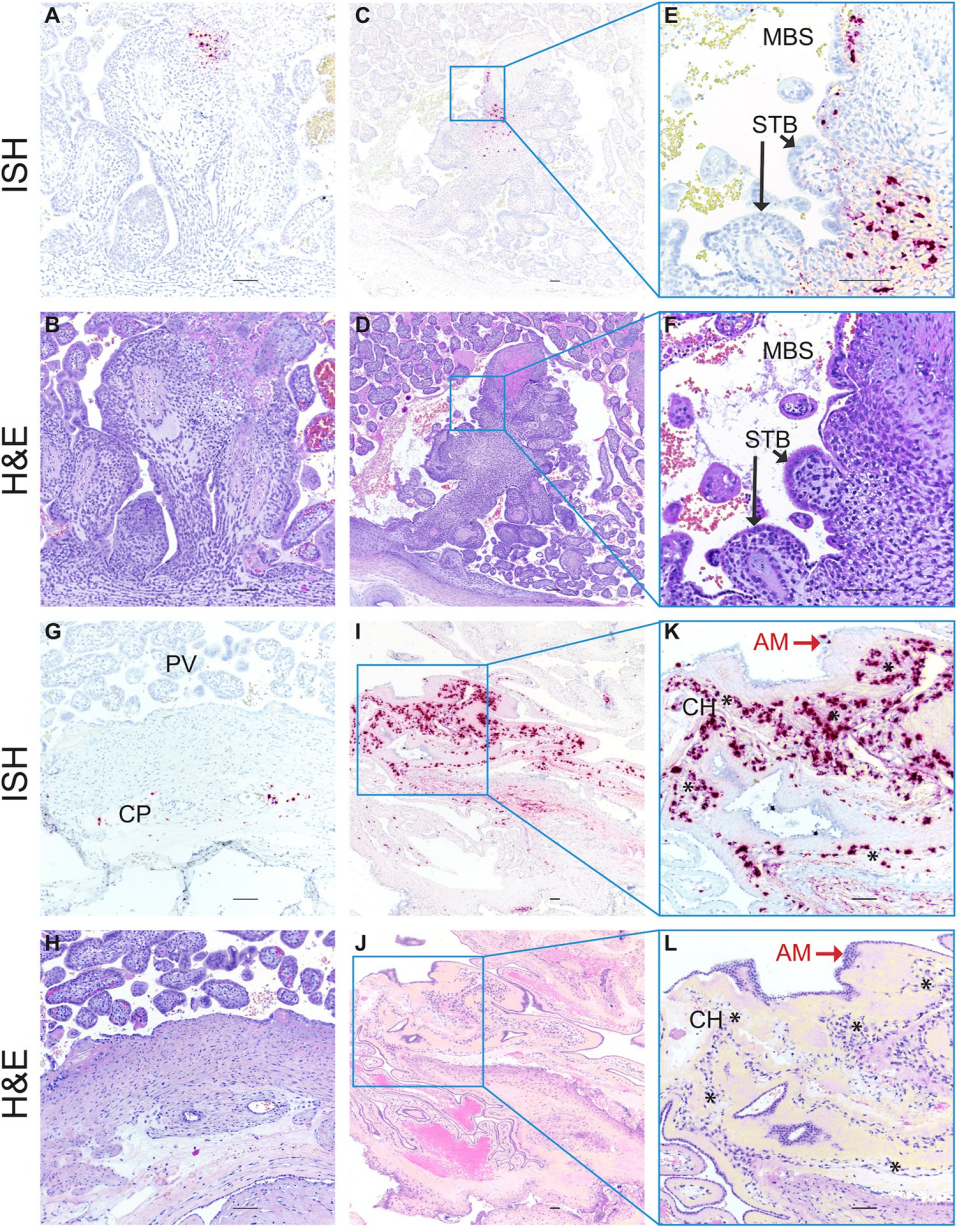
ZIKV RNA detected in MFI by *in situ* hybridization (ISH). Photomicrographs of paraffin embedded sections of the placenta and fetal membranes from 45/14-4. Panels A, C, E, G I, and K Positive ISH indicated by the red/pink chromogenic stain. Panels B, D, F, H, J, and L are corresponding H&E-stained sections to provide histological detail. Panels E, F K and J are higher magnification images of the corresponding regions in the adjacent photomicrographs. Scale bar represents 100 μm. Abbreviations: MBS maternal blood space, STB syncytiotrophoblasts, CP chorionic plate, PV placental villi, AM amniotic membrane, CH chorionic membrane. Black arrows indicate STB layer of placental villi in Panels E and F, red arrows indicate epithelial cells in the AM in Panels K and L, and * indicate the chorionic membrane layer of the fetal membranes in Panels K and L.

In the case of 45/14-4, where we have the most apparent evidence of vertical transmission to the fetus (Fig 4), ISH was performed on a full-thickness placental center cut. This pregnancy had a single disc placenta, an occurrence that happens in approximately 20% of rhesus macaque pregnancies [32, 33]. ISH evaluation of the placental center-cut showed ZIKV RNA in two large villi, one near the trophoblastic shell (Fig 6A and 6B) and the other near the chorionic plate (Fig 6C–6F). In both villi, the staining is located predominantly in the villous mesenchyme, and not in the outer syncytiotrophoblast (STB) layer that is directly exposed to maternal blood. Immunohistochemical staining of serial sections of this specimen suggested that ZIKV RNA in the villous near the trophoblastic shell (Fig 6A) may be associated with either macrophages or trophoblasts (S7A, S7B Fig), while the ZIKV RNA in the large villous near the chorionic plate (Fig 6E) localized with cytokeratin-positive trophoblasts (S7E Fig). Additionally, ZIKV RNA was detected sporadically in the chorionic plate surrounding large fetal blood vessels (Fig 6G and 6H).

For 45/14-4, we obtained a high-quality sample of the fused fetal membranes (membranes were not fused in the other eight ZIKV-exposed pregnancies). The fetal membranes consist of two components: the outer chorionic membrane, which is continuous with the chorionic plate of the placenta, and the amniotic membrane, the inner membrane that is closest to the fetus and is in contact with amniotic fluid (Fig 4A). While ZIKV was not detected by RT-qPCR, ISH revealed the abundant presence of ZIKV RNA in the fetal membranes (Fig 6I–6L). By comparing the ISH section to an H&E-stained serial section, we see that the ZIKV RNA is mainly detected in the chorionic membrane of the fetal membranes, with relatively fewer areas where ZIKV RNA is detected in the amniotic membrane (Fig 6I–6L). We confirmed that the ZIKV RNA in the fetal membranes represented replicating virus using mFISH that detects ZIKV replicative intermediates (S6 Fig). Immunohistochemical staining for the macrophage marker CD163 suggests that at least some of the cells with ZIKV RNA may be macrophages (S8 Fig). The relatively abundant presence of ZIKV RNA within the chorionic membrane and the chorionic plate suggests that ZIKV may gain access to the fetus through the chorionic membrane.

### ZIKV-DAK infection has no substantial effect on the placenta

To determine the effect of ZIKV-DAK on the development of the fetus, we conducted histological evaluations of the placenta and the fetus and compared our findings to gestational-age-matched controls. Histological evaluation of the placenta was performed by a board-certified pathologist who specializes in human placental pathology; this pathologist was only informed of the gestational age and remained blinded to all ZIKV RNA results and pregnancy treatment. The majority of the findings within an animal were either present or absent in both placental discs. Therefore, histopathological findings for the primary and secondary discs have been reported together (Table 1). The histological findings broken down by disc as well as placental weights and dimensions are provided in S2 Table.

**Table 1:** Histopathological evaluation of MFI tissue sections. The presence or absence of specific histological lesions in the MFI are indicated for mock-infected control pregnancies, and ZIKV-inoculated pregnancies. The criteria for acute villitis excluded interface necrosis in the villi anchored to maternal decidua.

Group	Treatment	Pregnancy	Gestational age (days)	Chronic deciduitis	Villitis	Maternal leukocy-toclastic decidual vasculitis	Maternal decidual necrosis	Acute villous infarction	Villous calcification
**30/7**	mock	30/7-C1	37	No	No	No	No	No	No
	ZIKV-DAK 7 dpi	30/7-1	38	No	No	No	**Yes**	No	No
		30/7-2	36	No	No	No	No	No	No
**30/14**	mock	30/14-C1	44	No	No	No	No	No	No
		30/14-C2	45	No	No	No	**Yes**	No	**Yes**
		30/14-C3	45	No	**Yes**	No	No	**Yes**	**Yes**
	ZIKV-DAK 14 dpi	30/14-1	43	No	No	No	**Yes**	No	No
		30/14-2	43	No	No	**Yes**	No	No	No
		30/14-3	45	No	No	No	**Yes**	No	**Yes**
**45/14**	mock	45/14-C1	58	**Yes**	No	No	No	No	No
		45/14-C2	60	No	No	No	No	No	No
		45/14-C3	61	No	No	No	No	**Yes**	No
	ZIKV-DAK 14 dpi	45/14-1	61	No	No	**Yes**	No	No	**Yes**
		45/14-2	60	No	**Yes**	**Yes**	**Yes**	**Yes**	**Yes**
		45/14-3	59	No	No	No	No	No	No
		45/14-4	63	No	No	No	No	**Yes**	No
Instances in mock				1	1	0	1	2	2
Instances in ZIKV-DAK				0	1	3	4	2	3
Total instances				1	2	3	5	4	5

Several of the pathologies that were found in the ZIKV specimens were also found in control specimens, suggesting that the majority of these lesions are not the result of ZIKV infection. We did see more instances of decidual necrosis in ZIKV cases compared to controls (Table 1), however this difference was not statistically significant (p = 0.3077). Decidual necrosis was seen in one out of the seven control cases and in four out the nine ZIKV cases. The decidual necrosis observed was focal. Additionally, decidual leukocytoclastic vasculitis was observed in the spiral arteries in three out of the nine ZIKV cases but not in any of the control cases, however this observed difference was not statistically significant (p = 0.2125). We found that decidual vasculitis was associated with the ZIKV RNA within decidual vessels. We observed more patchy nodular decidual necrosis in ZIKV cases (4/9) compared with controls (1/7) (Table 1), but there were areas of decidual necrosis in controls that make this feature less specific than vasculitis. IHC staining for endothelial cell marker CD31 suggests that the ZIKV RNA is within the endothelial cells in these vessels (S9E Fig). We also found that necrosis of anchoring villi in case 45/14-2 correlated with ZIKV infected macrophages (S10 Fig). Collectively, the histopathological evaluation of the placentas from control and ZIKV-exposed pregnancies suggest that ZIKV infection did not have any substantial effect on the placenta.

**Fig 7:**
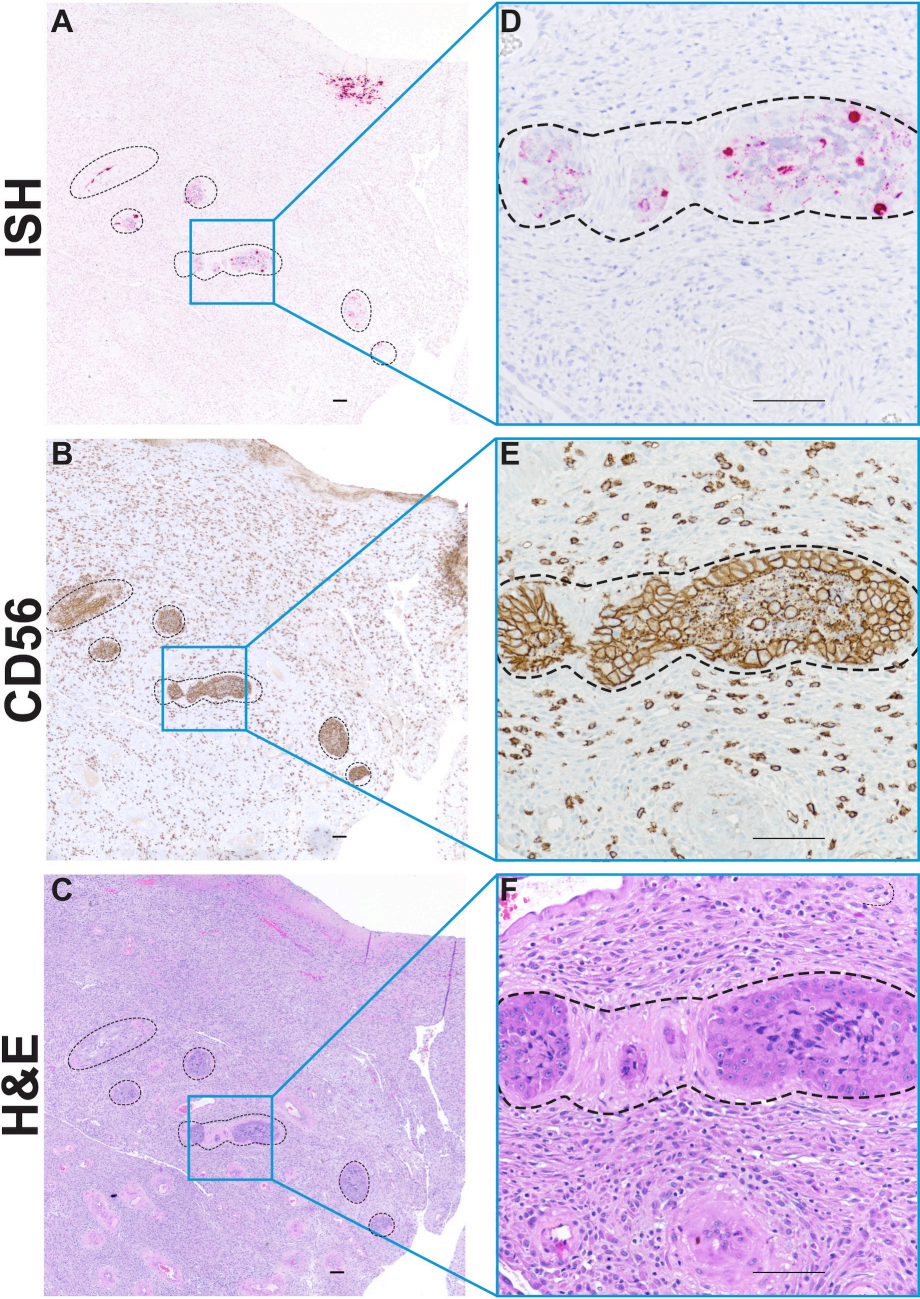
ZIKV RNA in decidual spiral arteries. Photomicrographs of paraffin embedded sections of the decidua basalis from 30/7-2. Spiral arteries are outlined by dashed lines. A) Pink staining shows ZIKV RNA detected using ISH in multiple profiles of a decidual spiral artery with invading luminal extravillous trophoblasts (EVTS). B) Immunohistochemical staining for CD56 reveals large EVT cells in outlined area, as well as smaller immune cells outside the outlined area. C) H&E stained serial section. Panels D, E and F represent higher magnification of images of indicated regions of Panels A, B, and C respectively. Scale bar represents 100 μm.

Importantly, we also found ZIKV RNA within decidual spiral arteries in case 30/7-2 (Fig 7). To determine what cell types ZIKV RNA was detected in, we performed IHC to identify extravillous trophoblasts (EVTs), endothelial cells, and macrophages (Fig 7E). IHC staining for CD56 revealed that endovascular EVTs were among the cell types that appeared to be infected with ZIKV. This finding is important because endovascular EVTs play an essential role in spiral artery remodeling [34, 35] and as seen in case 30/7-2 EVTs form “plugs” in early pregnancy that impact uteroplacental blood flow [36].

Histological evaluation of the fetus was performed by board-certified veterinary pathologists who were blinded to all RNA results. Eight out of the nine fetuses had no significant histologic lesions when compared to controls. The fetus with multiple ZIKV positive tissues, 45/14-4, had evidence of mild acute hemorrhage in the lung, but this lesion was presumed to be perimortem. Overall, there was no significant pathology in any of the ZIKV exposed fetuses. However, because all nine fetuses were terminated within 14 days of maternal inoculation, we are unable to evaluate what the effects of ZIKV infection in the fetal compartment might have been if the pregnancies had continued.

## Discussion

In this study, we inoculated nine pregnant rhesus macaques with 44 PFU of ZIKV-DAK early in pregnancy at either gd 30 or gd 45 with surgical pregnancy termination at 7 or 14 dpi. All dams became productively infected and developed strong neutralizing antibody responses. Evaluation of the MFI revealed ZIKV RNA in MFI tissues in all pregnancies. Three pregnancies also had infectious ZIKV in the amniotic fluid. One case had ZIKV RNA detected in multiple tissues in the fetus proper. ISH evaluation of MFI tissues showed that the decidua basalis was frequently infected and that the fetal membranes may play a role in ZIKV vertical transmission. No significant histologic pathology in the fetuses or the placenta was found. This study demonstrates that African-lineage ZIKV, like Asian-lineage ZIKV, can be vertically transmitted to the macaque fetus during pregnancy.

### Low-dose inoculation

Productive infection in all dams with inoculation of 44 PFU of ZIKV-DAK suggests maternal infection can occur with a relatively low inoculation dose in macaques. Furthermore, it demonstrates that maternal inoculation with a low dose can achieve vertical transmission, and that vertical transmission with African-lineage ZIKV is not simply a function of a higher dose inoculum as suggested by the comparison of two previously published studies [18, 21]. Vertical transmission was seen in the Raasch et al. study with an inoculation dose of 1 x 10^8^ PFU [21], but not seen in the Crooks et al. study with an inoculation dose of 1 x 10^4^ PFU (pregnancies evaluated at term). Styer et al. determined that West Nile virus-infected mosquitoes, when feeding on a live host, inoculate with a dose ranging from as low as approximately 5 PFU to as high as 10^6.6^ PFU, with a median dose ranging from 10^3.4^–10 ^6.1^ PFU depending on mosquito species [37]. Hence, 44 PFU is within the possible range of doses administered by a bite from an infected mosquito, but is likely far from a representative median dose. The low-dose inoculation used in this study suggests a low minimum infectious dose for rhesus macaques and may indicate a high epidemic potential of African ZIKV strains. Although the 44-PFU dose in the current study was unintentional, it has provided novel insight into permissiveness to ZIKV infection in pregnant macaques and risk factors for vertical transmission.

When comparing our data to a study by Crooks et al. [18] in which pregnant rhesus macaques were inoculated with 10^4^ PFU of ZIKV-DAK, we found that although our dams had a statistically significant delay (2.5 days) in the timing of peak viremia, viral RNA reached similar levels in the blood [18]. Additionally, the neutralizing antibody responses observed in the present study were similar to those reported by Crooks et al. [18]. These data suggest that infection with approximately 200 times less infectious virus did not drastically alter the maternal infection dynamics. However, we cannot determine the long-term impact of the inoculation dose on the fetus since pregnancies in this study were terminated within 14 dpi. Additionally, because of the novel nature of this study, we do not know if a comparably low dose inoculation in other animal models would result in in similar viral dynamics as it did in rhesus macaques. Thus, it is possible that the response to an inoculation with 44 PFU of ZIKV-DAK is a species-specific response.

### Vertical transmission

The current study suggests that this African-lineage ZIKV isolate, like Asian-lineage ZIKV, can be vertically transmitted to the fetus during pregnancy. ZIKV was vertically transmitted in three pregnancies, as evident by infectious virus in the amniotic fluid. The amniotic fluid is absorbed by the embryo/early fetus prior to skin keratinization [39], thus the embryo/early fetus has no barrier protecting it from infectious ZIKV in the amniotic fluid. Later in gestation the fetus also swallows amniotic fluid [38], this would be another way that ZIKV in the amniotic fluid can infect the fetus, however the fetuses in the current study have not yet developed the ability to swallow.

ZIKV RNA was also found in the fetus in two of those three pregnancies. Although the current study used a very small inoculation dose, our results are consistent with two other non-human primate studies that show that maternal infection with African-lineage ZIKV results in MFI infection and vertical transmission to the fetus [18, 19]. Consistent with the Crooks et al study [18], we did not observe any instances of fetal death or any fetal pathology. However, we did find evidence of vertical transmission in 3/9 pregnancies and pathology in the decidua. Two out of the three pregnancies that had vertical transmission were inoculated at gd 30, this is consistent with previously published studies demonstrating that ZIKV inoculation at gd 30 results in a high rate of vertical transmission compared to inoculation at gd 45 [39]. We saw vertical transmission in one of the cases inoculated at gd 45, this is somewhat unexpected as the Crooks study found that ZIKV-DAK inoculation at gd 34 with approximately 200 times more virus did not result in vertical transmission [18]. However, the pregnancies in Crooks study were evaluated 110–115 dpi, and evaluation of the fetus and MFI at 14 dpi in the current study likely improved our chances of detecting vertical transmission and histopathology that may have resolved if the pregnancies were allowed to continue to full term.

In our most apparent case of ZIKV vertical transmission to the fetus (45/14-4), we detected infectious virus in the amniotic fluid and ZIKV RNA in multiple fetal tissues. Focal presence of ZIKV was observed in placental villi, whereas widespread infection was observed in the fetal membranes. This case was the only one where we observed ZIKV in the placental villi by ISH. The morphological evaluation suggests that ZIKV RNA is not present in the outer STB layer but may be in villous macrophages and other cytotrophoblast cells. This pattern of ZIKV in the placenta fits the pattern reported in other *in vivo* studies that found ZIKV infection in the chorionic plate and in the placental villous mesenchyme, but not in the STB layer [18, 19, 40]. Importantly, ZIKV RNA was found in a large portion of the fetal membranes in this case, specifically within the chorionic membranes. These data suggest that the fetal membranes may act as a ZIKV reservoir and potentially as a route for vertical transmission; however, the broad significance of this observation remains unclear and unfortunately both fetal membranes (amniotic and chorionic) were not collected for the majority of cases. In the other two cases with infectious ZIKV in the amniotic fluid the amniotic membrane was not collected, and we did not detect ZIKV in the chorionic membrane. We note that in future studies, a more thorough sampling of all components of the MFI in is needed.

### Impact of ZIKV-DAK on fetal development and pregnancy outcomes

Studies in mice suggest that congenital infection with African-lineage ZIKV is more likely to cause fetal demise while Asian-lineage is more likely to cause congenital defects [16, 17]. We did not observe any cases of fetal demise, nor did we see any significant pathology in any of the fetuses. However, our study design (low-dose inoculum and early pregnancy termination) may not be ideal for studying adverse fetal outcomes such as fetal loss. Furthermore, the lack of fetal pathology hindered our ability to discern whether maternal inoculation at gd 30 versus gd 45 differentially affected the fetus.

We were able to observe ZIKV RNA within trophoblasts located within the lumina of maternal decidual spiral arteries. As far as we are aware, this is the first observation of ZIKV infection of endovascular trophoblasts *in situ* in decidual spiral arteries. Newman et al. reported persistent muscularization in spiral arteries in the decidua of a near-term rhesus macaque pregnancy vaginally challenged with ZIKV-DAK [19]. The importance of the remodeling of these vessels for the establishment of a successful pregnancy [41, 42] provides potential insight into the risk of adverse pregnancy outcomes with early pregnancy maternal ZIKV infection [7, 9].

Congenital birth defects or adverse pregnancy outcomes have not been formally associated with African-lineage ZIKV infection during pregnancy in people. However, African-lineage ZIKV is still considered and monitored as an emerging threat to public health [43]. Like Asian-lineage ZIKV, adverse pregnancy outcomes in people may only be detected when African strains of ZIKV emerge in a ZIKV-naive population. Recently, African-lineage has been reportedly found in Brazil, highlighting the risk of this virus to emerge outside the African continent [Preprints 14, 15]. African-lineage ZIKV has been shown to cause fetal demise in *in vivo* studies done in mice and macaques [17, 19], further supporting the potential of African-lineage ZIKV to cause adverse pregnancy outcomes in humans. The current study is in agreement with those findings.

In conclusion, this study demonstrates that African-lineage ZIKV, like Asian-lineage ZIKV, can be vertically transmitted from mother to fetus in the macaque model. It will be instructive to compare the localization of ZIKV at the MFI and in the fetus in these acute low-dose inoculations with parallel time point collections in pregnant rhesus monkeys receiving higher doses of ZIKV. Understanding how ZIKV compromises the “placental fortress” [44] will help map the pathway to vertical transmission of ZIKV. Understanding the pathway of ZIKV vertical transmission may better prepare researchers and clinicians for future viral outbreaks that may pose a risk for the health of the mother and developing fetus.

## Supporting information

S1 Table Summary of the timing of inoculation, and the days post infection (dpi) and gestational age at the time of pregnancy termination for each experimental subject. (TIFF)

S2 Table Histological evaluation broken down by placental disc. N/A = not applicable, placenta only had one disc. NR = not recorded, this measurement was not recorded.
(PNG)

S1 Fig Uninfected control placenta ZIKV RNA detection via ISH. Photomicrographs showing different regions of a placenta from an uninfected control pregnancy. A) Placental villi with decidua, B) Chorionic plate with placental villi, and C) higher magnification of image shown in panel B. D) Example of non-specific staining (pink) in the decidua and in E) placental villi. Staining that is limited to a single focus or with a non-cellular staining patterns was not interpreted as positive.
(TIF)

S2 Fig Timing and magnitude of peak viremia in dams inoculated with 44 PFU or 10^4^ PFU. A) Timing of peak viremia as measured by RT-qPCR of RNA isolated from plasma. Comparisons were made between dams inoculated with 44 PFU (n = 9) and dams inoculated with 10^4^ PFU (n = 4) (unpaired t-test, asterisk denotes p = 0.0150). B) Viral load on the day of peak viremia in dams inoculated with 44 PFU (n = 9) versus 10^4^ PFU (n = 4). The difference was not significant (unpaired t-test, P-value = 0.3729) The horizontal lines within the scatter plots represent the mean for each respective group. 10^4^ PFU data is from Crooks et al. [18].
(TIF)

S3 Fig Comparison of neutralizing antibody titers in pregnant rhesus macaques inoculated with 44 PFU vs 10^4^ PFU. A) PRNT_90_ values as measured in sera taken approximately 28 dpi from dams inoculated with 44 PFU (n = 7) versus 10^4^ PFU (n = 4). PRNT_90_ titers were not significantly different (unpaired t-test, P value = 0.1543). B) PRNT_50_ values as measured from serum taken approximately 28 dpi from dams inoculated with 44 PFU (n = 7) versus 10^4^ PFU (n = 4). PRNT_50_ titers were not significantly different (unpaired t-test, P value = 0.1253). The horizontal lines within the scatter plots represent the mean for each respective group. 10^4^ PFU data is from Crooks et al. [18].
(TIF)

S4 Fig Low levels of pre-existing immunity in 45/14-1. Plaque reduction neutralization tests (PRNT) were performed on serum samples collected prior to ZIKV inoculation. Data are expressed relative to infectivity in the absence of serum.
(TIFF)

S5 Fig Quantification of ZIKV RNA in maternal, MFI and fetal tissues. A) Viral loads determined by RT-qPCR of RNA isolated from biopsies taken from maternal tissues. B) tissue in the MFI. B) Viral loads determined by RT-qPCR of RNA isolated from biopsies taken from the MFI. C) ZIKV RNA burden in all maternal-fetal interface (MFI) tissues above the limit of detection from each group. Tissues include: decidua basalis, uterus-placental bed, placental biopsies, chorionic plate, chorionic membranes, amniotic membranes, and fused fetal membranes (only 45/14-4 had fused fetal membranes). The mean of each group is represented by the horizontal line. D) Viral loads determined by RT-qPCR of RNA isolated from umbilical cord and cord blood. Dashed lines represent the limit of detection.
(TIF)

S6 Fig ZIKV replicative intermediates detected in fetal membranes from 45/14-4 using multiplex fluorescence *in situ* hybridization (mFISH). Panels A, C, and E show different locations of the slide where ZIKV positive sense RNA (green) and negative sense RNA (red) were detected using mFISH. Nuclei of cells are stained with DAPI (blue). Panels B, D, and F show the ZIKV negative sense RNA detected alone in the same areas. Scale bars in A and E represent 20 μm and the scale bar in C represents 50 μm.
(TIF)

S7 Fig Immunohistochemical staining of ZIKV-positive placental villi from 45/14-4. Photomicrographs of paraffin embedded sections of the placenta from 45/14-4 (please refer to Fig 6A for ISH image). Cytokeratin staining in A), C) and E) identified trophoblasts cells. CD68 staining in B), D) and F) identify placental villous macrophages (Hoffbauer cells). E) and F) show a higher magnification of the corresponding regions of C) and D). Scale bars represent 100 μm.
(TIF)

S8 Fig Macrophages in the fetal membranes. A) photomicrographs of paraffin embedded sections of the fetal membranes from 45/14-4. A) Pink staining shows ZIKV RNA detected using ISH. B) Brown chromogen staining shows macrophages detected using immunohistochemistry staining for CD163. C) H&E stained serial section. Scale bars represent 100 μm.
(TIF)

S9 Fig Decidual vasculitis and ZIKV RNA in the decidua. Decidual vasculitis and ZIKV RNA in the decidua. Representative photomicrographs of paraffin embedded sections of the decidua with vasculitis from 30/14-2. A) and C) Pink staining shows ZIKV RNA detected using ISH in vessels associated with vasculitis. B) and D) H&E stained serial section. E) CD31 stained serial section. Lymphocytic infiltrate is indicated by asterisks. Scale bar represents 100 μm.
(TIF)

S10 Fig Necrosis in anchoring villi and ZIKV RNA in the trophoblastic shell. Representative photomicrographs of paraffin embedded sections of the trophoblastic shell with necrosis in the anchoring villi from 45/14-2. Area of necrosis is outlined by dashed line. A) Pink staining shows ZIKV RNA detected using ISH in the trophoblastic shell. B) H&E stained serial section. Scale bar represents 100 μm.
(TIF)
