# Supplementary figures and images for: Infection of the maternal-fetal interface and vertical transmission following low-dose inoculation of pregnant rhesus macaques (Macaca mulatta) with an African-lineage Zika virus
Source: PLoS One. 2023 May 4;18(5):e0284964. doi: 10.1371/journal.pone.0284964 (PMC10159132; doi:10.1371/journal.pone.0284964)

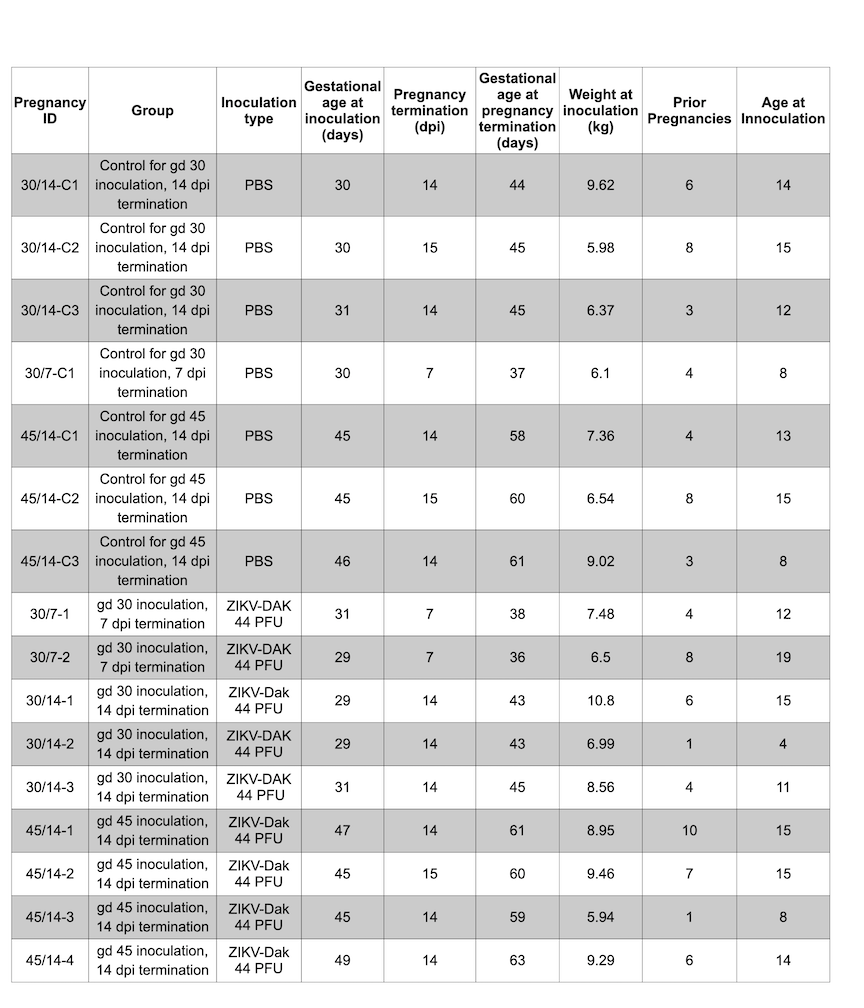

Supplement: S1 Table — (TIFF) [file pone.0284964.s001.tiff]

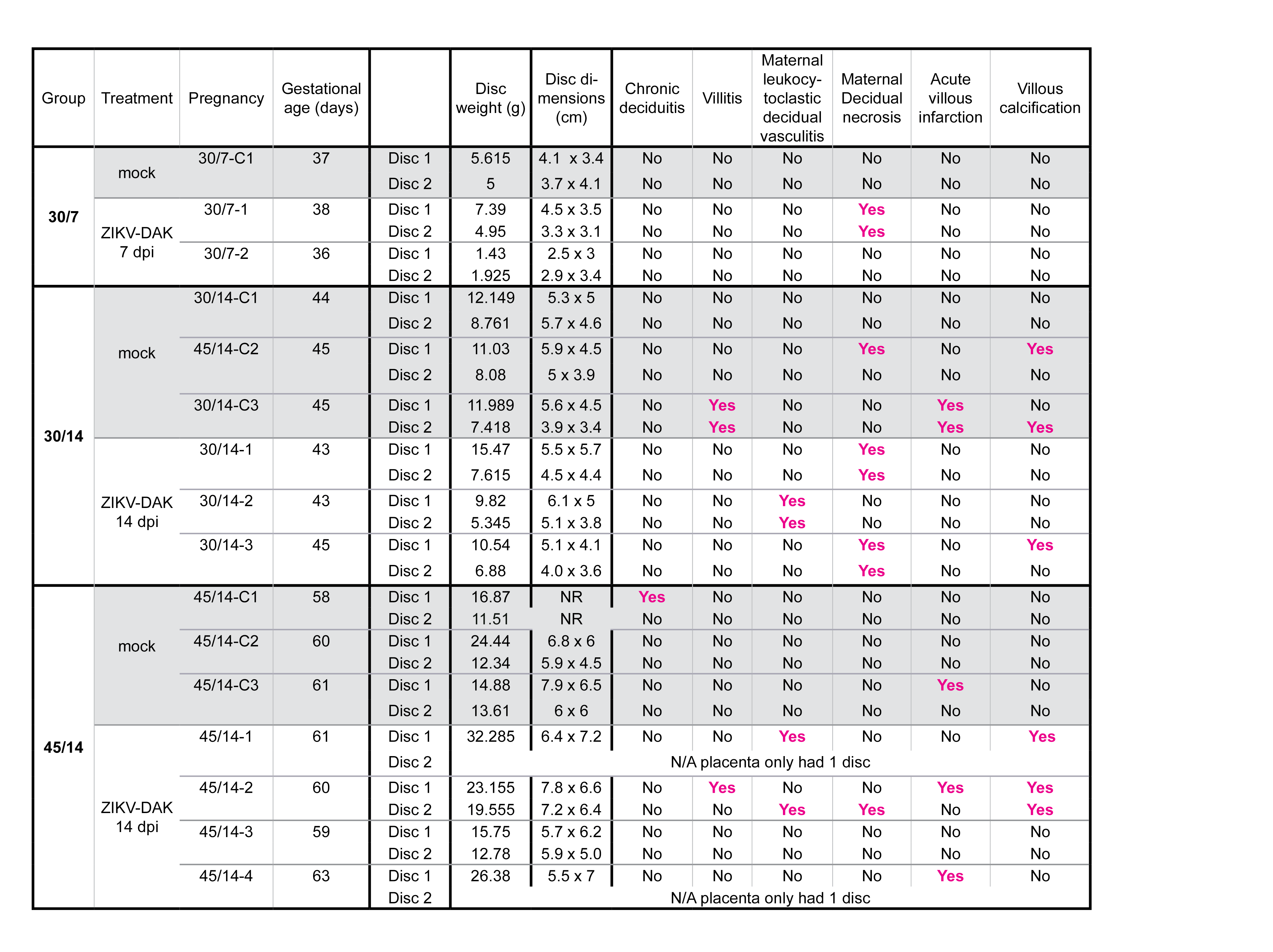

Supplement: S2 Table — N/A = not applicable, placenta only had one disc. NR = not recorded, this measurement was not recorded. (PNG) [file pone.0284964.s002.png]

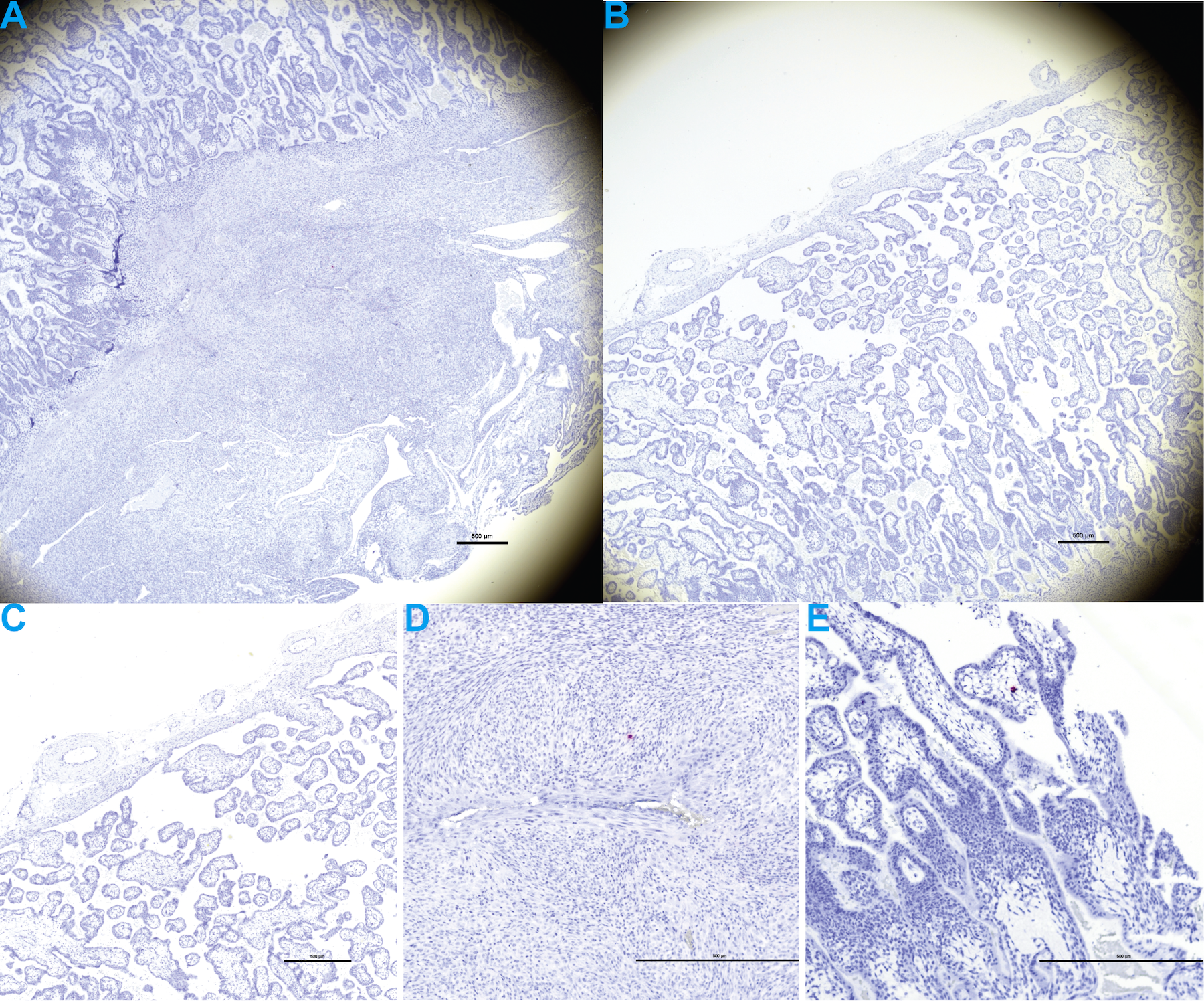

Supplement: S1 Fig — Photomicrographs showing different regions of a placenta from an uninfected control pregnancy. A) Placental villi with decidua, B) Chorionic plate with placental villi, and C) higher magnification of image shown in panel B. D) Example of non-specific staining (pink) in the decidua and in E) placental villi. Staining that is limited to a single focus or with a non-cellular staining patterns was not interpreted as positive. (TIF) [file pone.0284964.s003.tif]

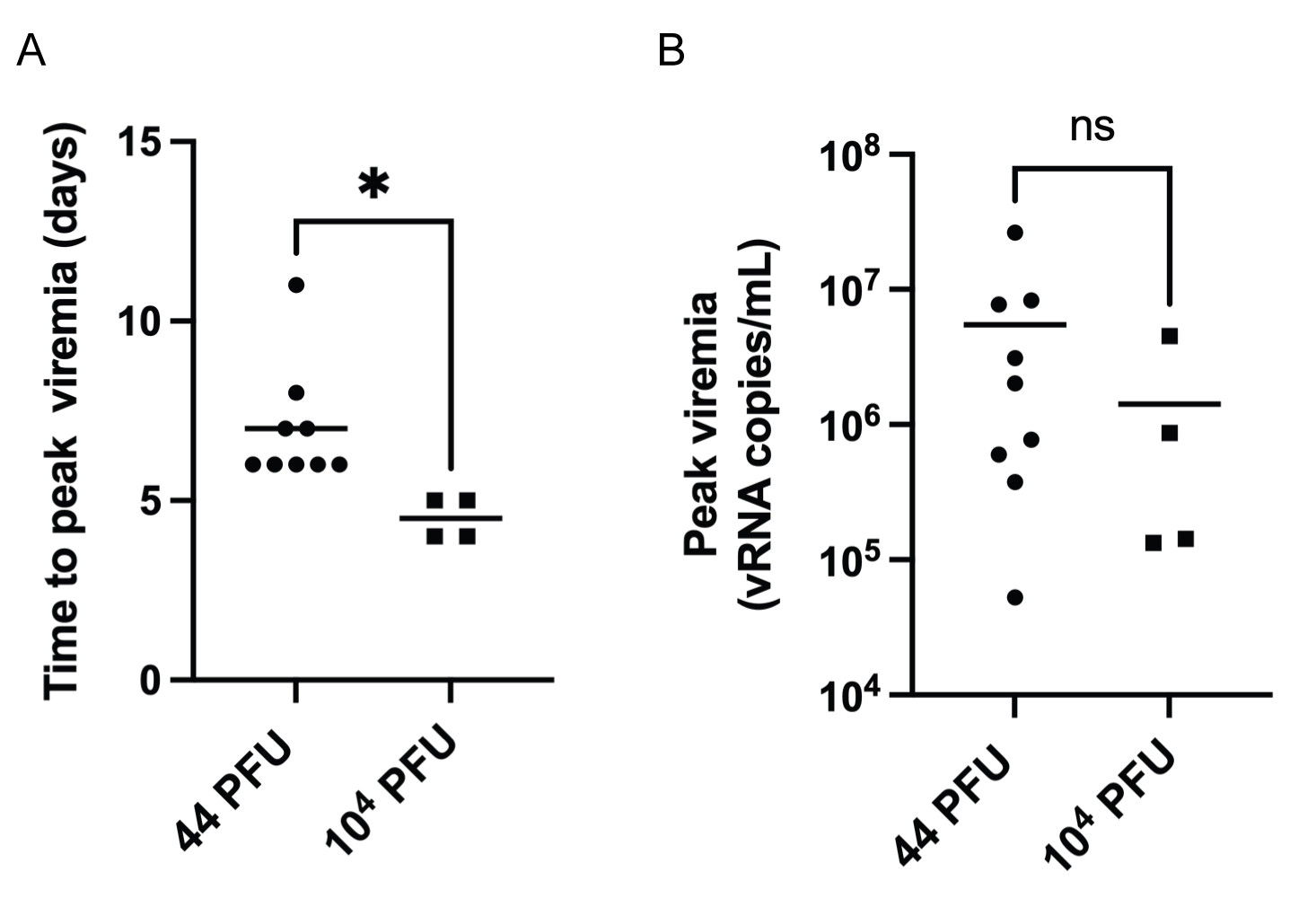

Supplement: S2 Fig — A) Timing of peak viremia as measured by RT-qPCR of RNA isolated from plasma. Comparisons were made between dams inoculated with 44 PFU (n = 9) and dams inoculated with 104 PFU (n = 4) (unpaired t-test, asterisk denotes p = 0.0150). B) Viral load on the day of peak viremia in dams inoculated with 44 PFU (n = 9) versus 104 PFU (n = 4). The difference was not significant (unpaired t-test, P-value = 0.3729) The horizontal lines within the scatter plots represent the mean for each respective group. 104 PFU data is from Crooks et al. [18]. (TIF) [file pone.0284964.s004.tif]

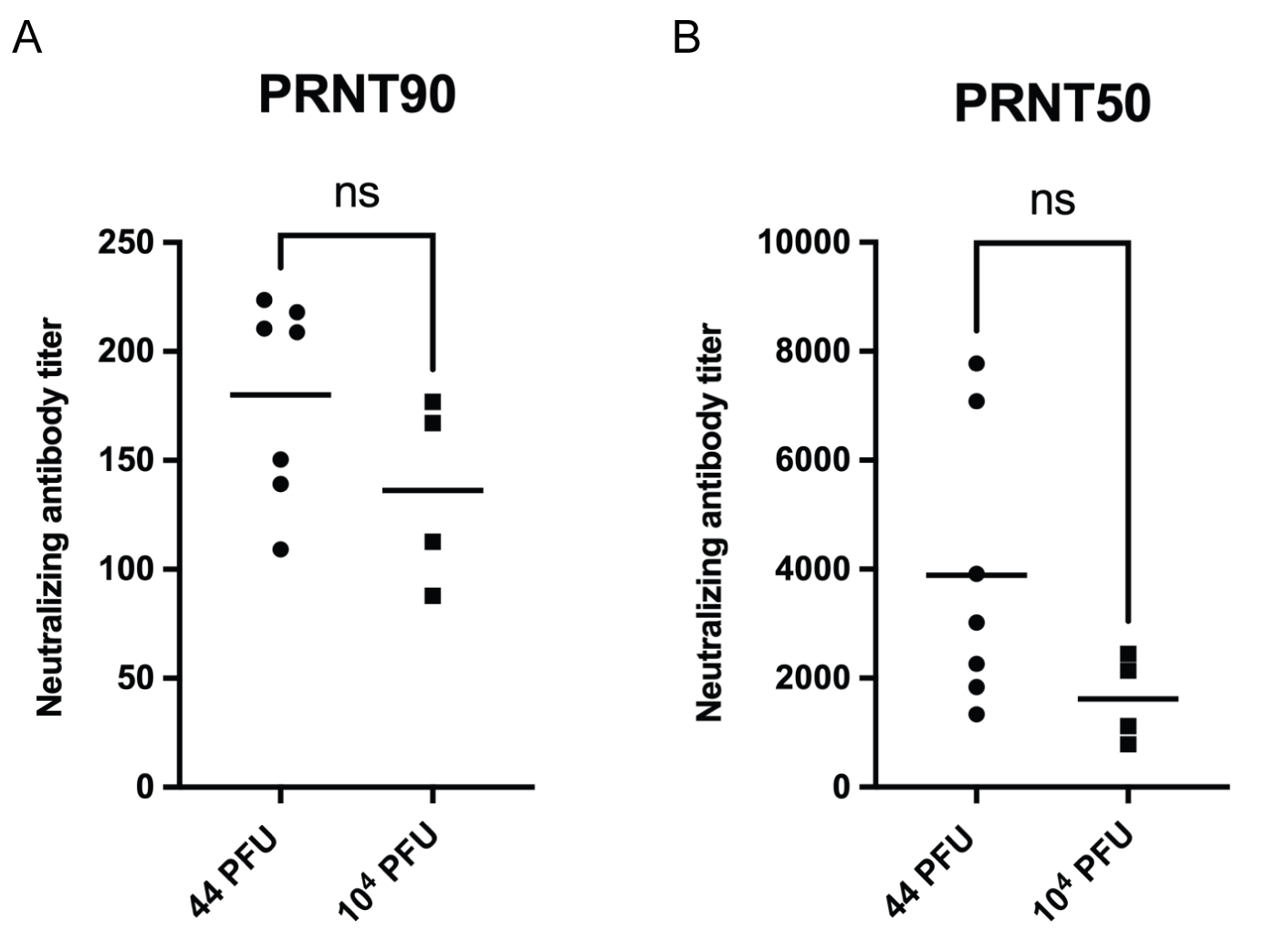

Supplement: S3 Fig — A) PRNT90 values as measured in sera taken approximately 28 dpi from dams inoculated with 44 PFU (n = 7) versus 104 PFU (n = 4). PRNT90 titers were not significantly different (unpaired t-test, P value = 0.1543). B) PRNT50 values as measured from serum taken approximately 28 dpi from dams inoculated with 44 PFU (n = 7) versus 104 PFU (n = 4). PRNT50 titers were not significantly different (unpaired t-test, P value = 0.1253). The horizontal lines within the scatter plots represent the mean for each respective group. 104 PFU data is from Crooks et al. [18]. (TIF) [file pone.0284964.s005.tif]

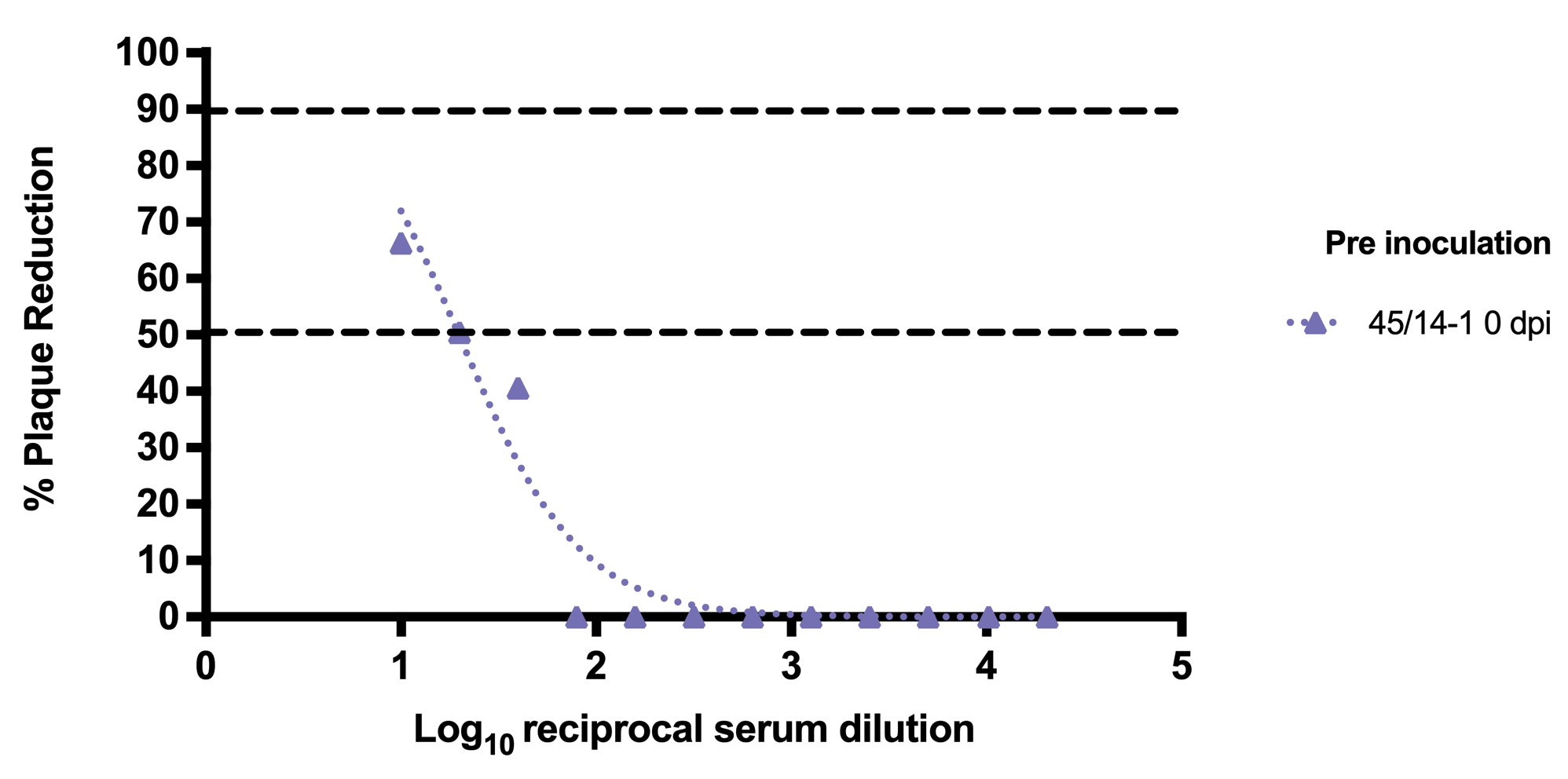

Supplement: S4 Fig — Plaque reduction neutralization tests (PRNT) were performed on serum samples collected prior to ZIKV inoculation. Data are expressed relative to infectivity in the absence of serum. (TIFF) [file pone.0284964.s006.tiff]

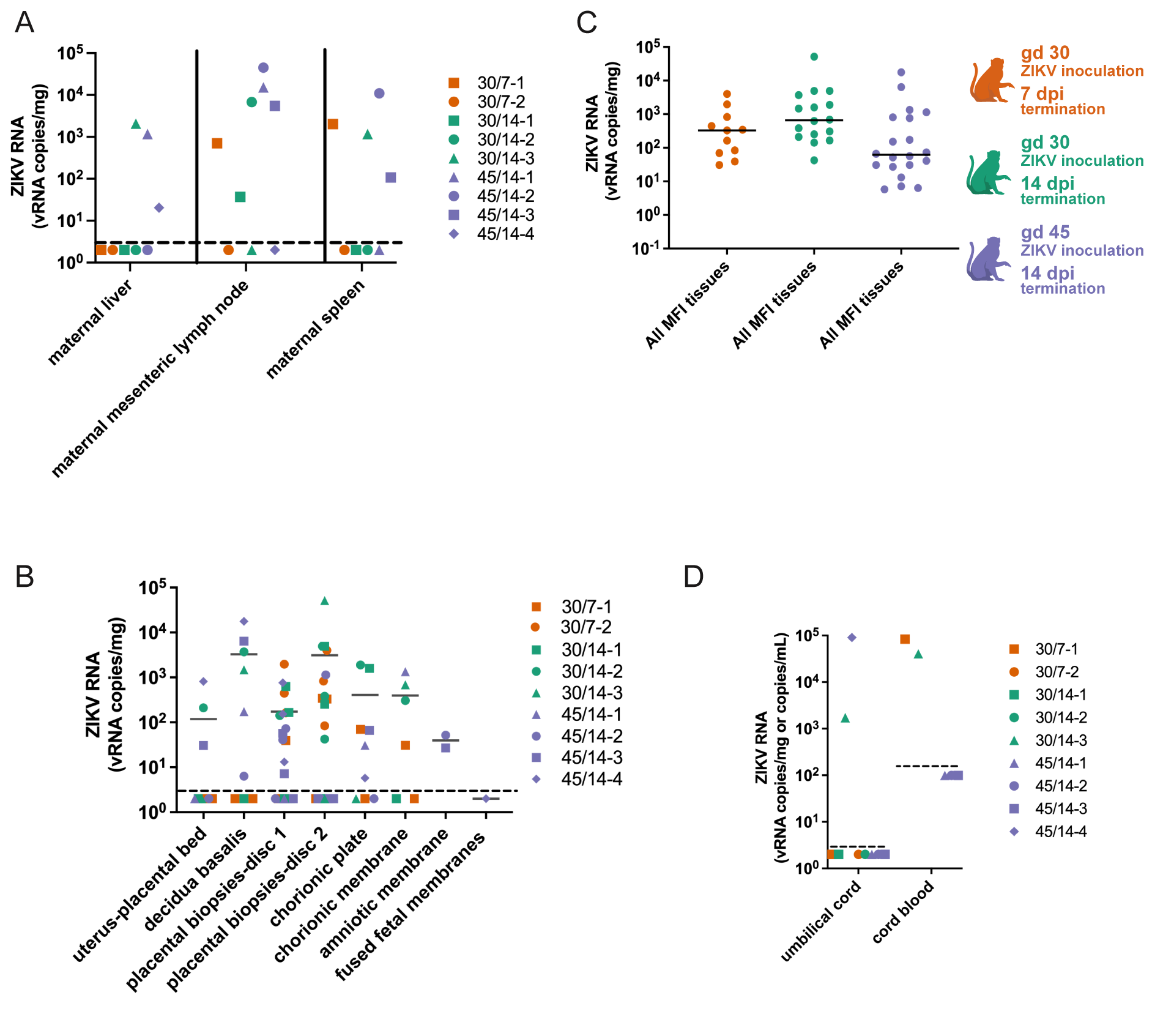

Supplement: S5 Fig — A) Viral loads determined by RT-qPCR of RNA isolated from biopsies taken from maternal tissues. B) tissue in the MFI. B) Viral loads determined by RT-qPCR of RNA isolated from biopsies taken from the MFI. C) ZIKV RNA burden in all maternal-fetal interface (MFI) tissues above the limit of detection from each group. Tissues include: decidua basalis, uterus-placental bed, placental biopsies, chorionic plate, chorionic membranes, amniotic membranes, and fused fetal membranes (only 45/14-4 had fused fetal membranes). The mean of each group is represented by the horizontal line. D) Viral loads determined by RT-qPCR of RNA isolated from umbilical cord and cord blood. Dashed lines represent the limit of detection. (TIF) [file pone.0284964.s007.tif]

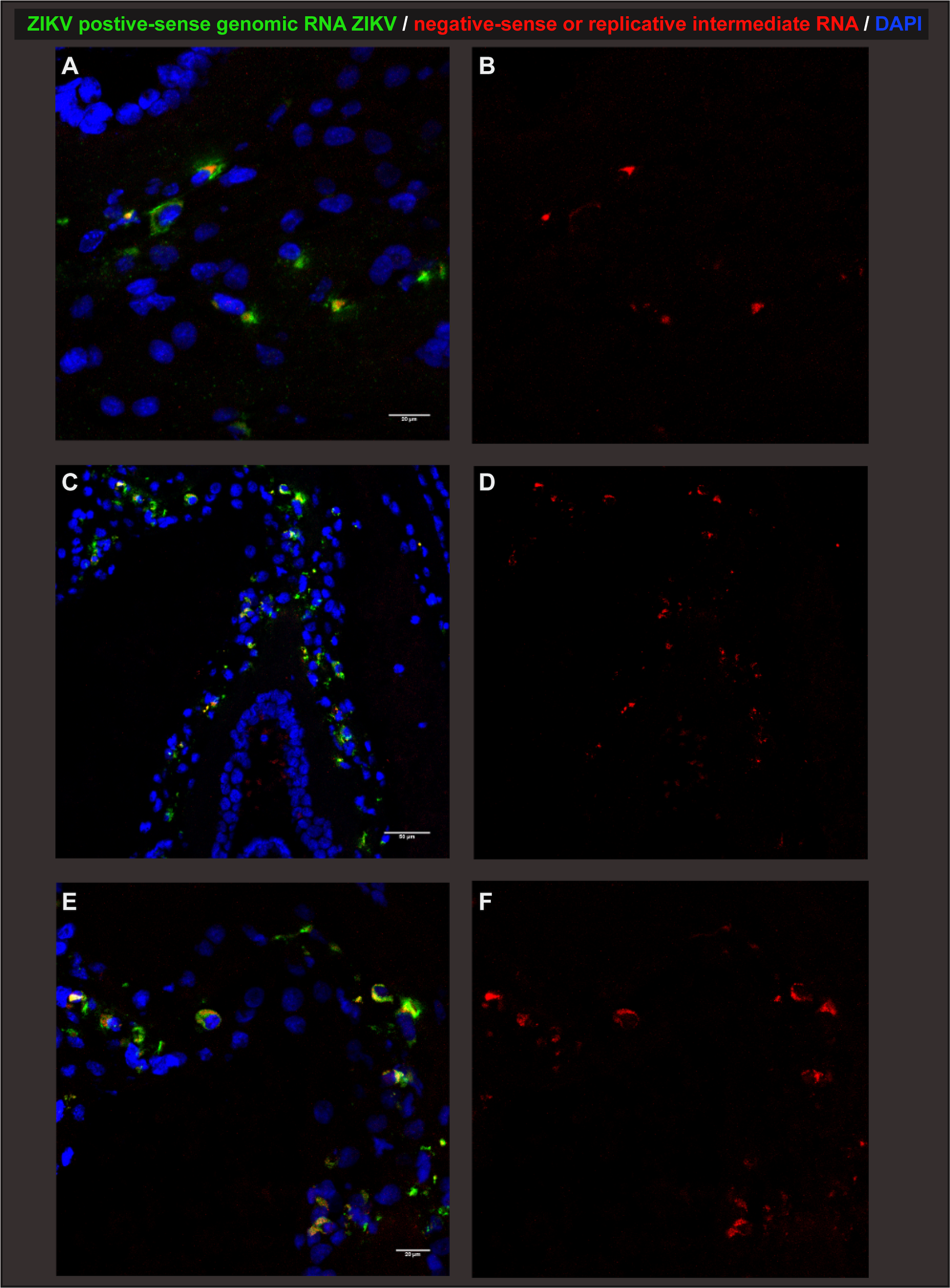

Supplement: S6 Fig — Panels A, C, and E show different locations of the slide where ZIKV positive sense RNA (green) and negative sense RNA (red) were detected using mFISH. Nuclei of cells are stained with DAPI (blue). Panels B, D, and F show the ZIKV negative sense RNA detected alone in the same areas. Scale bars in A and E represent 20 μm and the scale bar in C represents 50 μm. (TIF) [file pone.0284964.s008.tif]

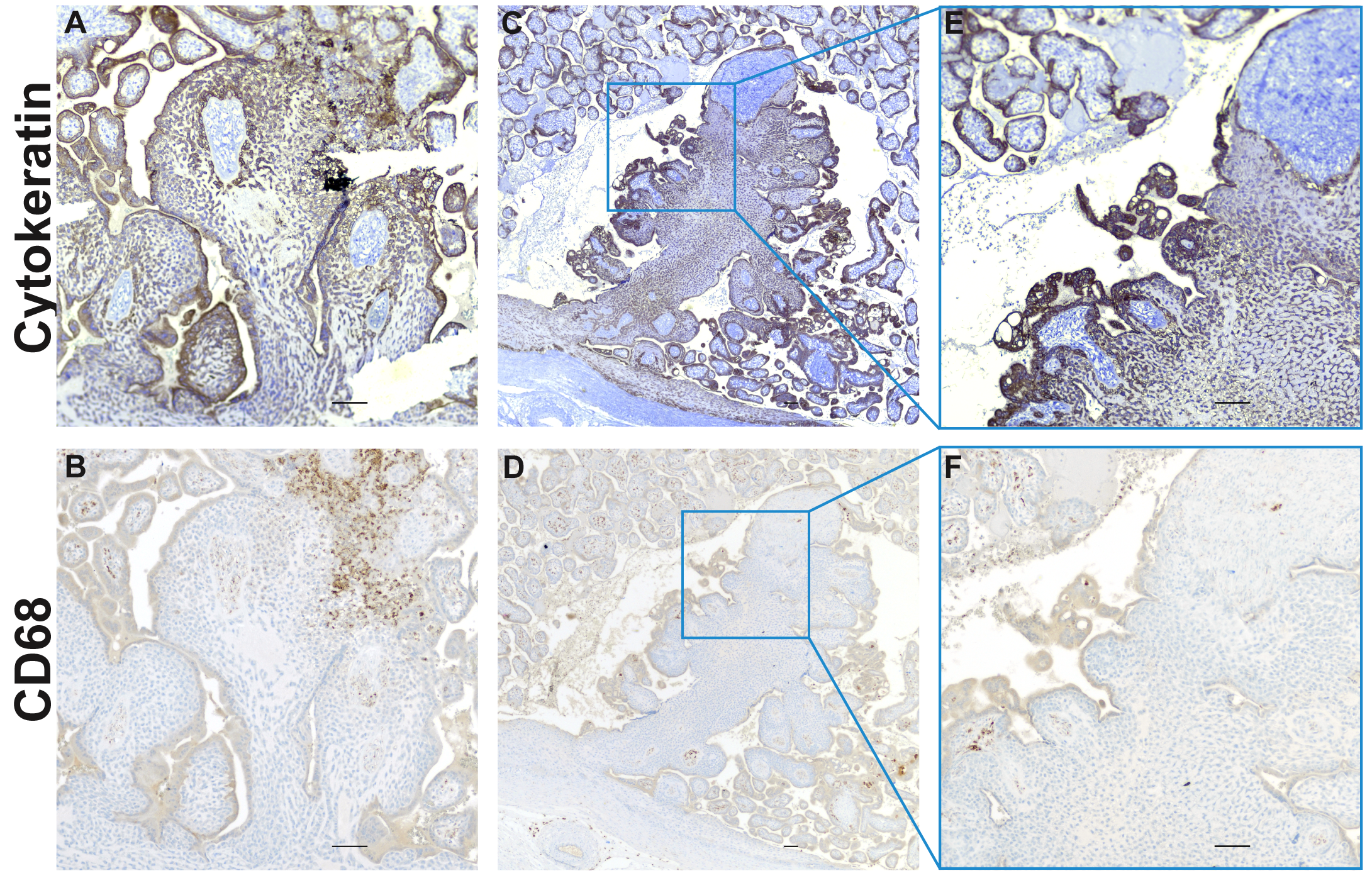

Supplement: S7 Fig — Photomicrographs of paraffin embedded sections of the placenta from 45/14-4 (please refer to Fig 6A for ISH image). Cytokeratin staining in A), C) and E) identified trophoblasts cells. CD68 staining in B), D) and F) identify placental villous macrophages (Hoffbauer cells). E) and F) show a higher magnification of the corresponding regions of C) and D). Scale bars represent 100 μm. (TIF) [file pone.0284964.s009.tif]

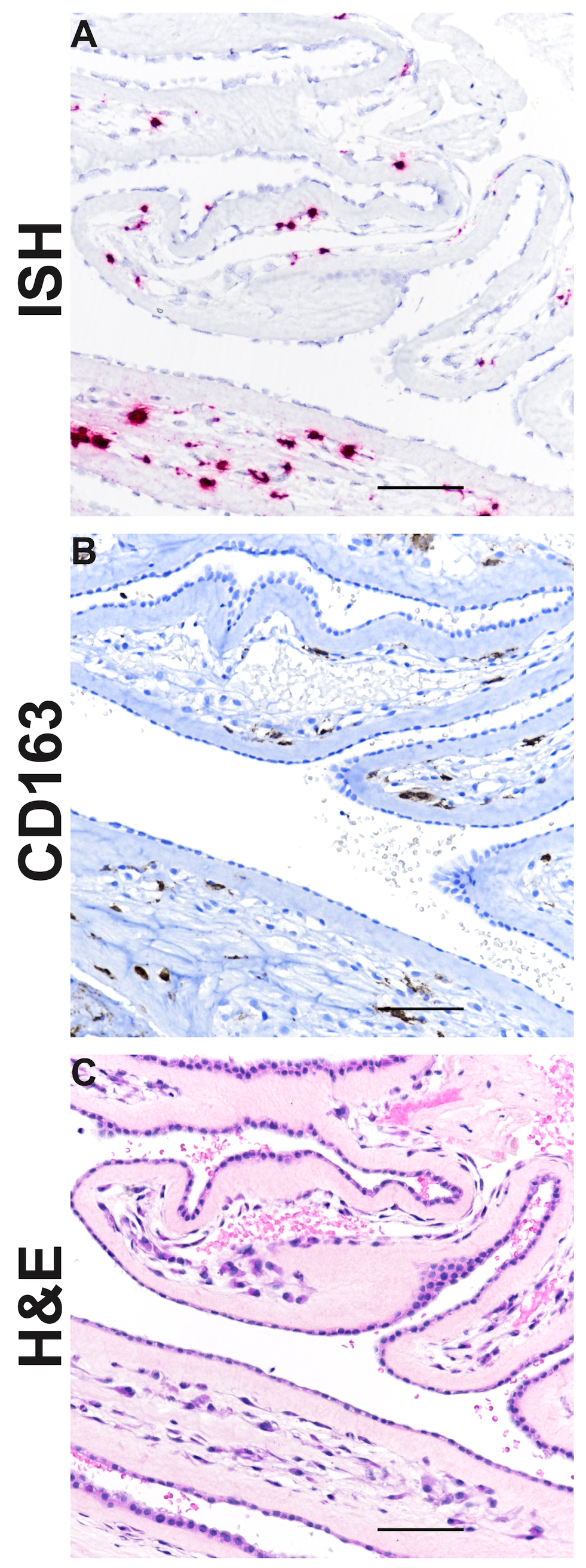

Supplement: S8 Fig — A) photomicrographs of paraffin embedded sections of the fetal membranes from 45/14-4. A) Pink staining shows ZIKV RNA detected using ISH. B) Brown chromogen staining shows macrophages detected using immunohistochemistry staining for CD163. C) H&E stained serial section. Scale bars represent 100 μm. (TIF) [file pone.0284964.s010.tif]

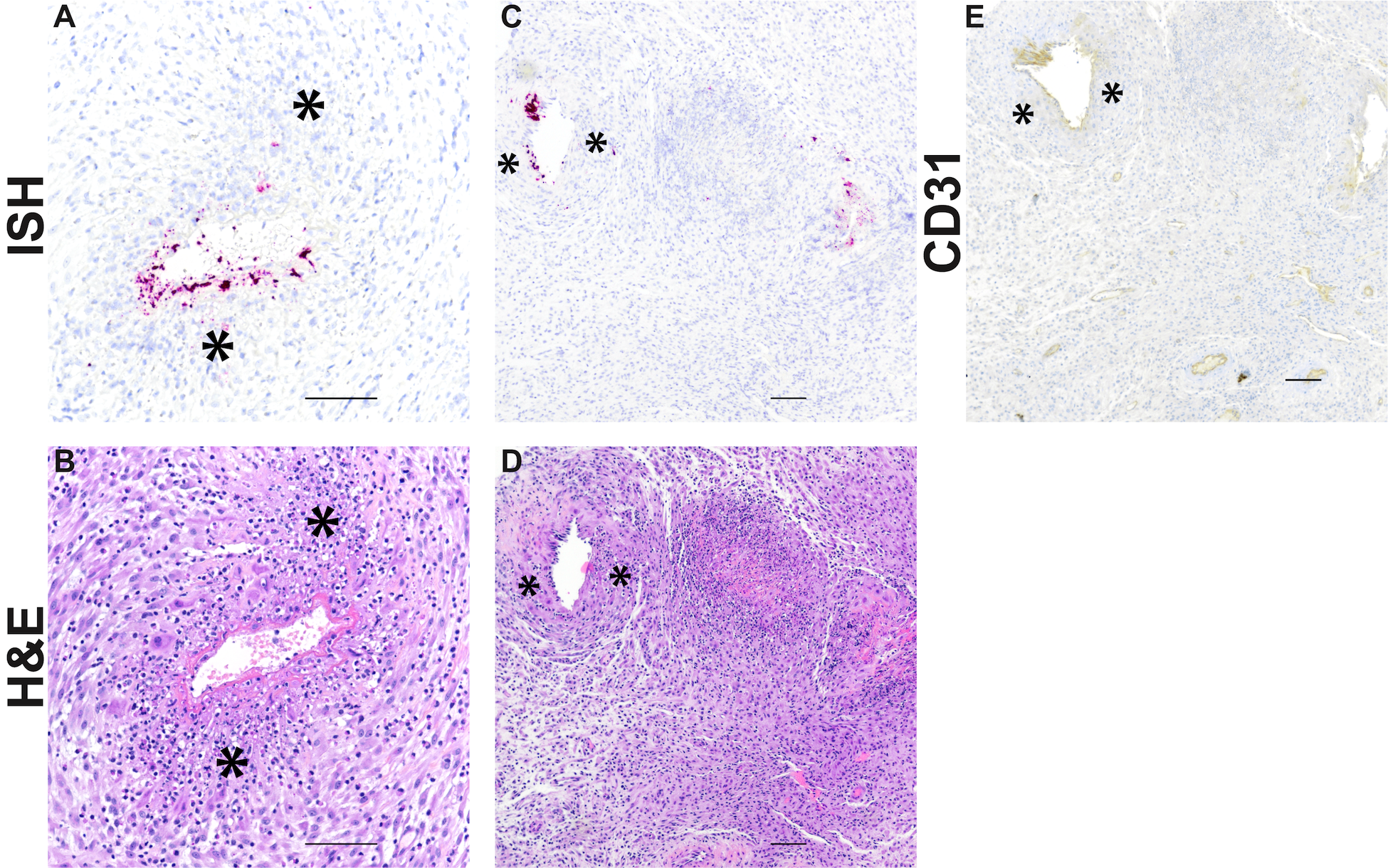

Supplement: S9 Fig — Decidual vasculitis and ZIKV RNA in the decidua. Representative photomicrographs of paraffin embedded sections of the decidua with vasculitis from 30/14-2. A) and C) Pink staining shows ZIKV RNA detected using ISH in vessels associated with vasculitis. B) and D) H&E stained serial section. E) CD31 stained serial section. Lymphocytic infiltrate is indicated by asterisks. Scale bar represents 100 μm. (TIF) [file pone.0284964.s011.tif]

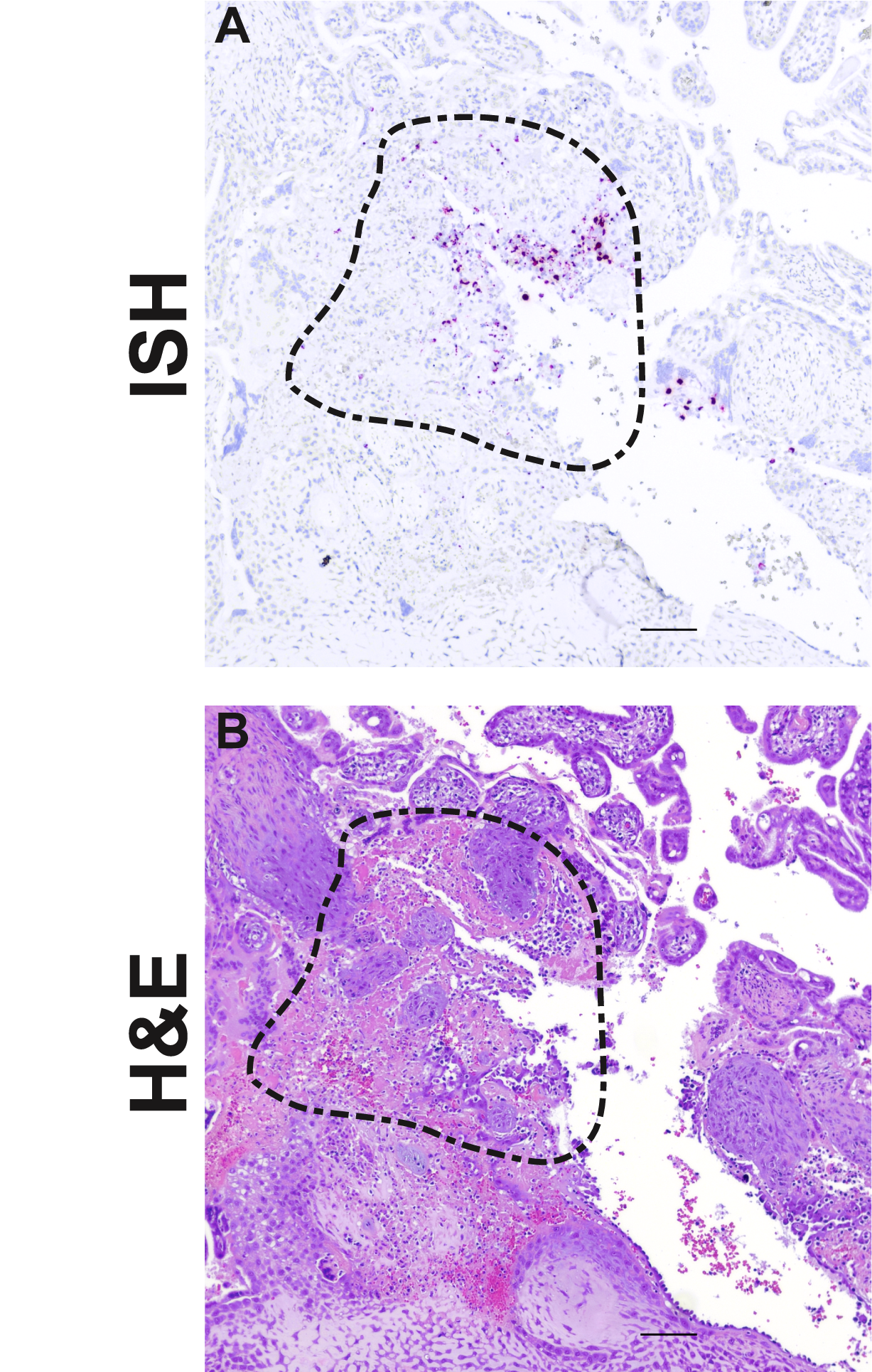

Supplement: S10 Fig — Representative photomicrographs of paraffin embedded sections of the trophoblastic shell with necrosis in the anchoring villi from 45/14-2. Area of necrosis is outlined by dashed line. A) Pink staining shows ZIKV RNA detected using ISH in the trophoblastic shell. B) H&E stained serial section. Scale bar represents 100 μm. (TIF) [file pone.0284964.s012.tif]
